# α-cell electrophysiology and the regulation of glucagon secretion

**DOI:** 10.1530/JOE-22-0295

**Published:** 2023-06-26

**Authors:** Rui Gao, Samuel Acreman, Jinfang Ma, Fernando Abdulkader, Anna Wendt, Quan Zhang

**Affiliations:** 1 Oxford Centre for Diabetes, Endocrinology and Metabolism, University of Oxford, Churchill Hospital, Oxford, UK; 2 Department of Physiology, Institute of Neuroscience and Physiology, Metabolic Research Unit, University of Gothenburg, Göteborg, Sweden; 3 Department of Physiology and Biophysics, Institute of Biomedical Sciences, University of Sao Paulo, Sao Paulo, Brazil; 4 Department of Clinical Sciences Malmö, Islet Cell Exocytosis, Lund University Diabetes Centre, Lund University, Malmö, Sweden; 5 CNC - Center for Neuroscience and Cell Biology, CIBB - Centre for Innovative Biomedicine and Biotechnology, University of Coimbra, Coimbra, Portugal

**Keywords:** α-cell, electrophysiology, glucagon, diabetes

## Abstract

Glucagon is the principal glucose-elevating hormone that forms the first-line defence against hypoglycaemia. Along with insulin, glucagon also plays a key role in maintaining systemic glucose homeostasis. The cells that secrete glucagon, pancreatic α-cells, are electrically excitable cells and use electrical activity to couple its hormone secretion to changes in ambient glucose levels. Exactly how glucose regulates α-cells has been a topic of debate for decades but it is clear that electrical signals generated by the cells play an important role in glucagon secretory response. Decades of studies have already revealed the key players involved in the generation of these electrical signals and possible mechanisms controlling them to tune glucagon release. This has offered the opportunity to fully understand the enigmatic α-cell physiology. In this review, we describe the current knowledge on cellular electrophysiology and factors regulating excitability, glucose sensing, and glucagon secretion. We also discuss α-cell pathophysiology and the perspective of addressing glucagon secretory defects in diabetes for developing better diabetes treatment, which bears the hope of eliminating hypoglycaemia as a clinical problem in diabetes care.

## Introduction

In the body, blood glucose levels are kept within a narrow range by concerted action of glucagon and insulin, the glucose-regulating hormones secreted by α- and β-cells, respectively (Göke 2008). Whereas the islet α-cells were identified histologically already in 1907 (by then they were named ‘A-cells’) (Lane 1907), the hormone they produce, glucagon, was discovered in 1923 and described as a ‘glucose agonist’ that can rapidly increase blood glucose (Kimball & Murlin 1923), an effect opposite to that of insulin. Because of its potent glucose-elevating efficacy, glucagon plays a key role in glucose counter-regulation against hypoglycaemia and has been used as an emergency antidote for severe hypoglycaemia (Elrick *et al.* 1958), a life-threatening medical condition of dangerously low blood glucose that is often caused by iatrogenic use of insulin/insulin secretagogues.

Normally, glucagon secretion is stimulated by a fall in blood glucose but suppressed at euglycaemia and hyperglycaemia (Göke 2008). This secretory pattern becomes defective in diabetes and leads to glycaemic volatility: failure to secrete glucagon at low glucose contributes to the occurrence of hypoglycaemia (Cryer & Gerich 1985), while hyperglucagonemia at high glucose exacerbates hyperglycaemia (Menge *et al.* 2011). Therefore, therapies that can restore normal α-cell function/glucagon secretion would significantly improve diabetes treatment, particularly for better prevention of hypoglycaemia. However, exactly how α-cells function becomes dysregulated in diabetes remains unknown. This gap in the knowledge is mainly because the normal cellular mechanism that regulates α-cells remains incompletely understood, despite over five decades of research.

Like β-cells, α-cells are excitable cells and couple electrical activity to glucagon release; but different from β-cells, α-cells are electrically active at low glucose (Zhang *et al.* 2013). Although there are various (and sometimes opposite!) theories on how glucose metabolism regulates α-cell activity (Gylfe 2016), it is clear that the electrophysiological control of α-cells plays an important role in their nutrient sensing and glucagon secretion. The development of improved patch-clamping techniques (Hamill *et al.* 1981) has decisively boosted the understanding of the ion currents associated with α-cell excitability (Wesslen *et al.* 1987, Rorsman & Hellman 1988) and glucagon secretion (Gromada *et al.* 1997). The current state of the art on this interplay between α-cell electrophysiology and glucagon secretion is the scope of this review, where we attempt to provide an overview that may help to understand how these elusive cells function and, importantly, how these regulatory mechanisms may become defective in diabetes.

## A brief history of α-cells

**Figure 1:**
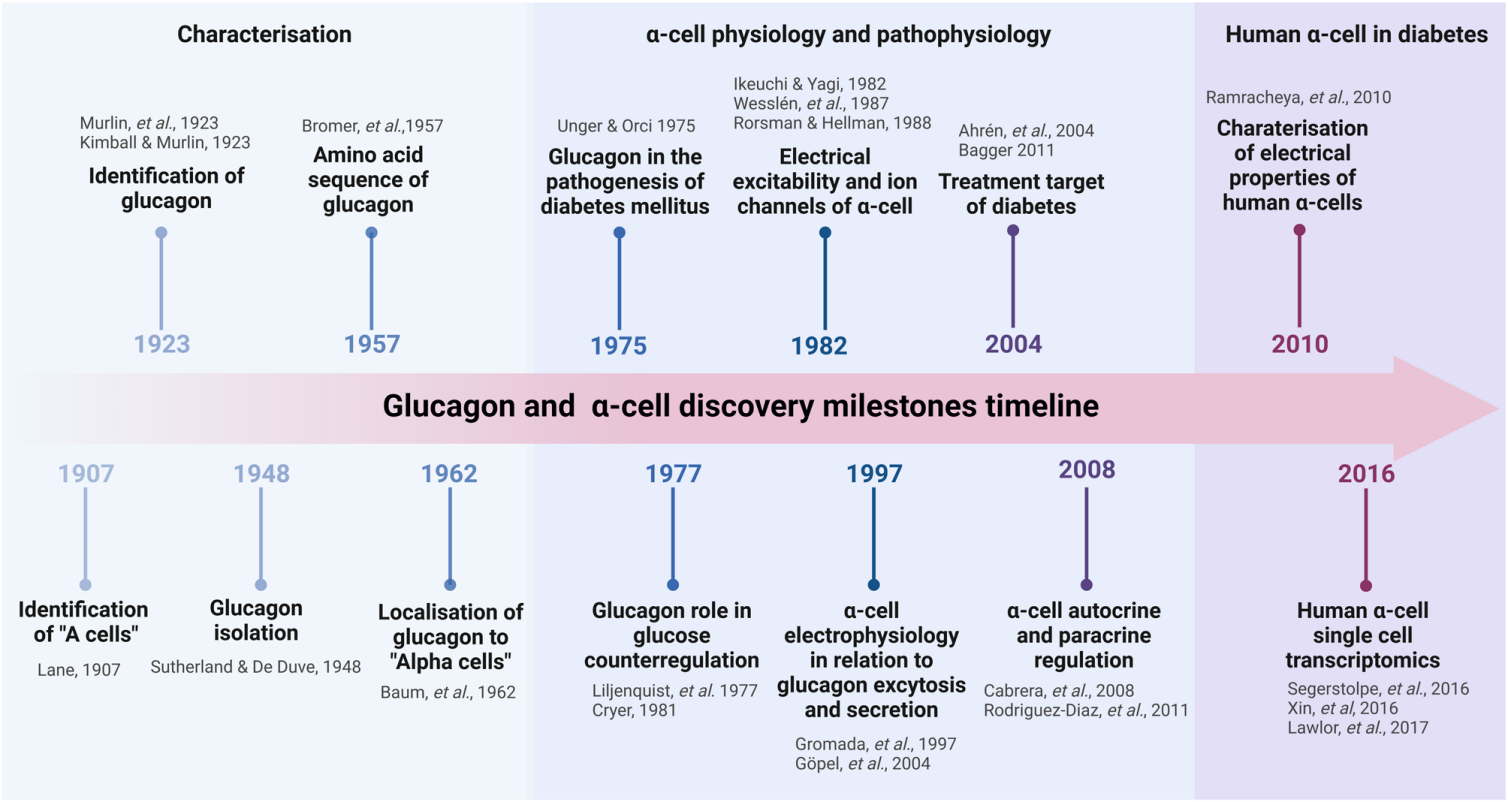
Glucagon and α-cell discovery milestone timeline. The three phases of glucagon and α-cell research history with bullet points referring to references in the text. Created with BioRender.com.

Although glucagon has not received as much attention as insulin, its history is almost as long (Fig. 1, a simplified timeline). The first description of a pancreatic factor that raised blood glucose concentration was made a century ago, in 1923, when Murlin and colleagues discovered a fraction in a pancreatic extract that had ‘the power to act in just the opposite way to insulin; namely, to raise the blood sugar…’ (Murlin *et al.* 1923), which later they named glucagon(Kimball & Murlin 1923). However, the actual isolation of the glucagon molecule only happened in 1948 (Sutherland & De Duve 1948) and its crystallisation in 1953 (Staub *et al.* 1953). This was quickly followed by its clinical applications in the 1950s to reverse hypoglycaemia (Elrick *et al.* 1958). The development of the indirect immunofluorescence technique by Coons *et al.* (Coons *et al.* 1955) and the production of reliable glucagon antibodies by Unger *et al.* (Unger *et al.* 1959) were pivotal for Baum *et al.* to localise glucagon expression to ‘alpha cells’ (Baum *et al.* 1962), the cells that were histologically identified in 1907 by maintaining their staining properties upon ‘alcohol’ fixation (‘A cells’) (Lane 1907), comfiring the biochemical observation made by Sutherland and De Duve (Sutherland & De Duve 1948). The glucagon antibodies also led to the development of a radioimmunoassay (Unger *et al.* 1959) which measures glucagon, enabling accurate evaluation of glucagon secretory responses.

But how do α-cells work? They were shown to be electrically excitable (Ikeuchi & Yagi 1982), and historical milestones related to α-cell electrophysiology include the development of experimental approaches using intact islets to study α-cell ionic currents (Göpel *et al.* 2000), exocytosis (Göpel *et al.* 2004), and electrical activity and the electrophysiological characterisation of human α-cells (Ramracheya *et al.* 2010).

The increasing availability of human pancreatic islets for research from the beginning of the 21st century has revolutionised our understanding of the α-cell. As Dolenšek *et al.* pointed out, there are remarkable morphological differences between rodent and human endocrine pancreas that must have functional implications (Dolenšek *et al.* 2015). Indeed, studies in human islets have shown that glucagon is not the only molecule secreted by α-cells with intra-islet signalling properties. It was elegantly demonstrated that glutamate is also secreted by human α-cells, upon which it has a stimulatory autocrine effect via AMPA/kainate ionotropic receptors (Cabrera *et al.* 2008). It has been suggested that human α-cells also secrete acetylcholine, which has a positive paracrine effect on neighbouring β-cells (Rodriguez-Diaz *et al.* 2011*b*). However, in a more recent study, vesicular acetylcholine transporters were found to be absent in human α-cells (Tang *et al.* 2018), echoing the transcriptomic data acquired in human islets (Xin *et al.* 2016). It will be possible to address this discrepancy by direct amperometric detection of acetylcholine release (Keighron *et al.* 2015) from α-cells at single-cell resolution. In addition, glucagon-like peptide 1 (GLP-1) was also found in a subset of human α-cells (Marchetti *et al.* 2012) and not only in intestinal L-cells. Interestingly, whereas GLP-1 content in non-diabetic human islets is only a fraction of that of glucagon (<1%) (Galvin *et al.* 2021), its secretion was significantly higher in islets from type 2 diabetic (T2D) donors than from euglycaemic donors, indicating that there may be a switch not only in glucose sensitivity but also in α-cell qualitative hormonal output in diabetes. Clearly, there is much more to the α-cell than meets the (glucagon) eye!

## Morphological considerations

In most living animals, hypoglycaemia is an uncommon and harmful condition that can be corrected by behavioural or hormonal regulation (counter-regulation). The release of counter-regulatory hormones (including glucagon) must be fast to avoid prolonged fuel deprivation for normal body function. Indeed, as a first-line defence against hypoglycaemia, glucagon secretion is rapid in response to hypoglycaemia (Schwartz *et al.* 1987) and is facilitated by several α-cell morphological features.

In many species (with the exception of guinea pig (Rorsman & Hellman 1988)), α-cells, compared to β-cells, are not only a relatively small population (~30–40%) but also smaller in size (diameter = 10 μm vs 15 μm in β-cells, in murine islets) (Barg *et al.* 2000). In electrophysiological terms, the small membrane area of α-cells determines their low membrane capacitance (~3 pF vs >5 pF in β-cells, conversion factor = 10 fF/μm^2^). Together with their spherical morphology (which enables rapid distribution of charges), minute changes in α-cell ion channel activity can quickly alter its whole-cell membrane potential (e.g. the velocity of action potential upstroke, d*V*/d*t*, reaches up to 37 V/s) (Zhang *et al.* 2013) and subsequent glucagon release.

Glucagon is released from α-cells through Ca^2+^-dependent exocytosis, a process where glucagon-containing granules fuse to the plasma membrane to release their cargo (Barg *et al.* 2000). α-cells are densely granulated with ~7000 granules per cell (i.e. the granular density is ~9 granules/μm^3^, given the average α-cell volume of ~800 μm^3^), occupying the majority of the cellular volume (average granule volume = 0.08 mm^3^) (Barg *et al.* 2000). Therefore, many granules are docked at the plasma membrane (docked-granule density = 0.6 granules/μm^2^ (Omar-Hmeadi *et al.* 2020), accounting for ~180 docked granules per cell at a given time) and can be readily released upon stimulation. A single depolarisation as short as 20 ms is sufficient to trigger the release of ~50 granules (Hamilton *et al.* 2018). The high granule density may also contribute to a rapid refilling of the ‘readily-releasable pool’ of granules, enabling continuous high-speed exocytosis under repetitive stimulation (Barg *et al.* 2000). In addition, α-cells have an extensive endoplasmic reticulum (ER) network (Pfeifer *et al.* 2015) and can provide additional Ca^2+^ through the process of Ca^2+^-induced Ca^2+^ release (CICR) for high-volume exocytosis. This forms part of the adrenaline-stimulated glucagon secretion (Hamilton *et al.* 2018)

Together, these morphological features enable rapid and robust glucagon secretory response to hypoglycaemia, fulfilling its role as an emergency counter-regulatory hormone.

## Ion channels in α-cell electrical excitability, glucose sensing and glucagon secretion

### Voltage-gated ion channels

There is a plethora of ion channels expressed in α-cells, governing their cellular excitability and exocytosis (see illustration in Fig. 4). Like all excitable cells, α-cell electrical activity depends on the activity of its voltage-gated ion channels.

Voltage-gated Na^+^ channels (Na_v_ channels) are Na^+^-permeable pores that can be opened by membrane depolarisation. An influx of Na^+^ ions via Na_v_ channels can rapidly charge the membrane, forming/accelerating the upstroke of action potentials (APs), often leading to overshooting APs (peak potential >0 mV). Once opened, Na_v_ channels quickly (~2 ms) become inactivated and are only reactivated when the cell returns to resting membrane potential (Milescu *et al.* 2008). In α-cells, the total Na^+^ current is on average ~–450 pA (triggered by depolarisation from –70 mV to 0 mV) at the physiological range of membrane potential. Blocking Na_v_ channels with the broad-spectrum Na_v_ channel blocker tetrodotoxin reduces both α-cell AP amplitude and glucagon secretion at low glucose (Zhang *et al.* 2014).

**Figure 2:**
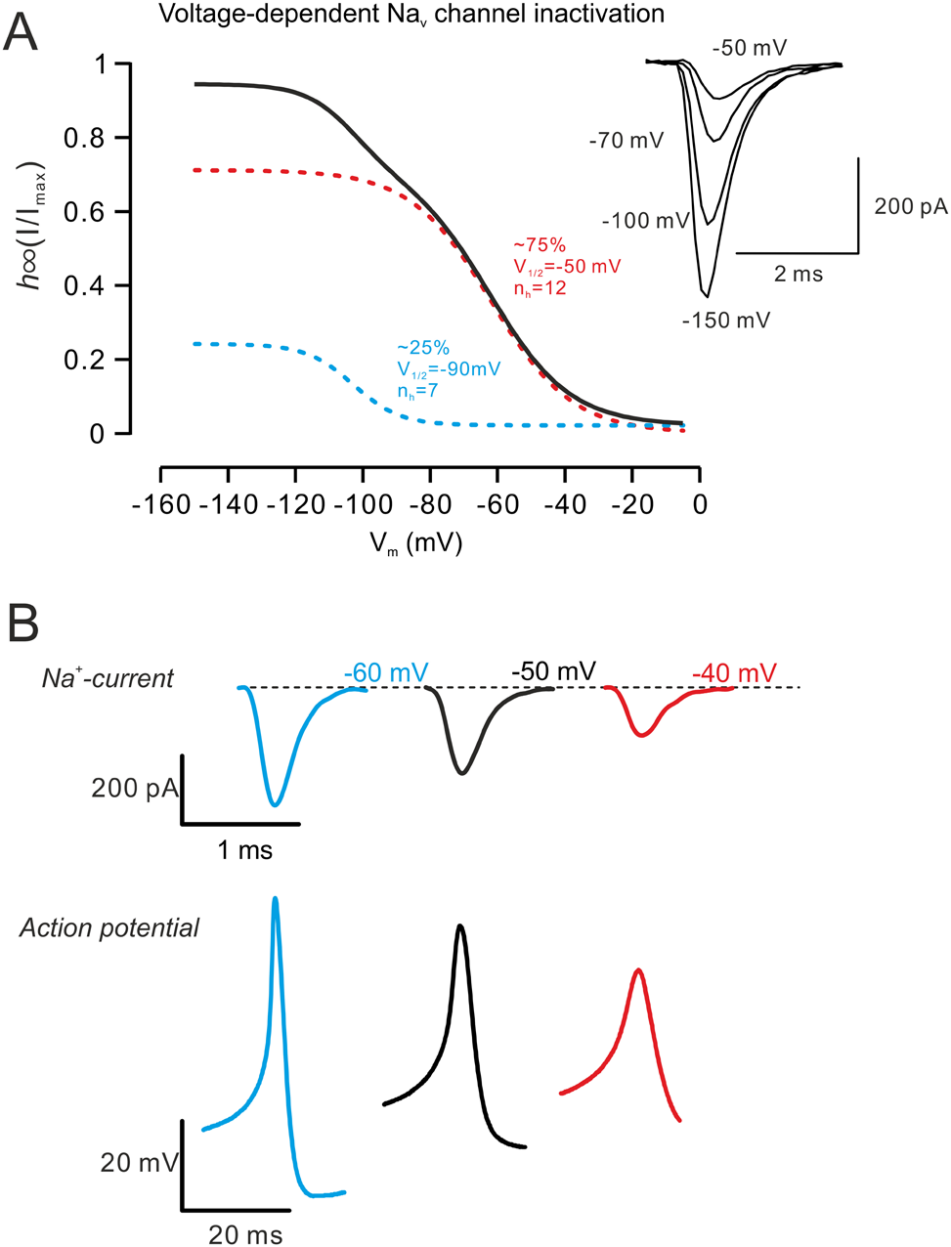
α-cell Na_v_ channels and action potentials. (A) Biphasic voltage-dependent inactivation of α-cell Na_v_ current (black), in which ~25% of the current (blue-dashed line) inactivates at negative voltage with a slow slope factor and ~75% (red-dashed line) inactivates at positive voltage with a fast slope factor. Examples of Na_v_ currents recorded at different membrane potentials are shown on the right. *h*_∞_, voltage-dependent inactivation; *I*_max_, total activatable Na^+^ current; *V*_1/2_, half maximal inactivation voltage; *n*_h_, slow factor of voltage-dependent inactivation. (B) Na_v_ currents and action potentials at different membrane potentials. Large Na^+^ current at negative membrane potential contributes to fast and high-amplitude α-cell action potential (blue traces), and small Na^+^ current at more positive membrane potential leads to low-amplitude α-cell action potential (red traces).

α-cell Na^+^ current mainly flows through two types of Na_v_ channels, with 70–90% of current flowing via Na_v_1.3 and 10–20% via Na_v_1.7 (Zhang *et al.* 2014). Therefore, the voltage-dependent inactivation of α-cell Na^+^ currents is biphasic: ~25% (the Na_v_1.7 component) inactivates half maximally (*V*_1/2_) at ~–90 mV, while the *V*_1/2_ of ~75% of the current (the Na_v_1.3 component) is ~–50 mV (Fig. 2A). Importantly, the slope of the voltage-dependent inactivation of the latter component is fast (*n*_h_=~12 mV), and as the membrane potential steps more positively, the number of activatable Na_v_ channels declines rapidly, reaching ~10% at ~–40 mV. Consequently, α-cell APs vary significantly at different membrane potentials, both in amplitude and in upstroke velocity (Fig. 2B). This has a direct impact on the activity of voltage-gated Ca^2+^ channels (Ca_v_ channels), the ion channels that provide exocytosis-triggering Ca^2+^ signals (Zhang *et al.* 2013).

Rodent α-cells are equipped with at least three types of high voltage-activated (HVA) Ca_v_ channels (L-, P/Q-, and N-type Ca_v_ channels) (Göpel *et al.* 2004, MacDonald *et al.* 2007, Zhang *et al.* 2013). Although the majority of the Ca^2+^ current flows through L-type Ca_v_ channels, blockade of these channels does not affect glucagon secretion/α-cell exocytosis in the absence of adrenergic activation, at least in mouse islets (Göpel *et al.* 2004). Instead, N‐ or P/Q‐type Ca_v_ channels are the exocytosis‐relevant Ca^2+^ channels in α-cells, despite their relatively low contribution to the transmembrane Ca^2+^ currents (~20% each) (De Marinis *et al.* 2010, Zhang *et al.* 2013). It is possible that α‐cells are compartmentalised, and N‐ and/or P/Q‐type Ca_v_ channels are tightly coupled to the exocytotic machinery, forming efficient exocytosis ‘hotspots’ (Xia *et al.* 2007). L-type Ca_v_ channels, on the other hand, may not be associated with these ‘hotspots,’ and the Ca^2+^ influx through these channels may bear other functions. Interestingly, the relationship between Ca_v_ channels and α-cell exocytosis/glucagon secretion becomes different in the presence of adrenaline, which activates β-adrenergic receptor to increase intracellular cAMP levels. Blocking L-type Ca_v_ channels with isradipine abolishes adrenaline-stimulated glucagon secretion and α-cell exocytosis, which are resistant to pharmacological-inhibition of P/Q-type (with ω-agatoxin VIA) or N-type Ca_v_ channels (ω-conotoxin GVIA) (Gromada *et al.* 1997). It is possible that high levels of cAMP enhance L-type Ca_v_ channel activity and recruit more granules for exocytosis. Furthermore, these channels may be coupled to ER Ca^2+^ release promoted by cAMP-dependent signalling pathways: (i) they may be linked with ER Ca^2+^ loading when α-cells are electrically active – blocking L-type Ca_v_ channels prior to adrenaline application attenuated adrenaline-induced increase in cytosolic Ca^2+^ (Hamilton *et al.* 2018), while acute application of the blocker only modestly reduced the effect of adrenaline. This may be in synergy with the store-operated ER-filling mechanism that is independent of α-cell electrical activity, which was elegantly demonstrated by the Gylfe group (Liu *et al.* 2004) and (ii) larger L-type Ca^2+^ current may activate ER Ca^2+^-releasing ryanodine receptors (Nordenskjöld *et al.* 2020) to directly induce ER Ca^2+^ release. Adrenaline can also stimulate Ca^2+^ release from α-cell ER via InsP_3_ receptors (InsP_3_Rs), activated by α1-adrenoceptor-generated InsP_3_ (Vieira *et al.* 2004). Indeed, blockade of the InsP_3_Rs with xestospongin C also reduced adrenaline-evoked α-cell Ca^2+^ increase (Hamilton *et al.* 2018). However, it is important to point out that the above observations were made in rodent islets, and the ion channel composition is quite different in human α-cells. Human islet electrophysiology studies pioneered by Braun and colleagues found that the contribution of transmembrane Ca^2+^ in human α-cells is almost opposite to that of the rodent α-cells, with 70% and 21% of whole-cell Ca^2+^ charge influx flowing through the P/Q-type and L-type Ca_v_ channels, respectively (the N-type Ca_v_ channels are responsible for the remaining ~10%) (Ramracheya *et al.* 2010). It should be noted that this does not completely reflect the amplitude of the Ca^2+^ currents that flow through the two HVA Ca_v_ channels, since they have distinct channel kinetics (L-type Ca_v_ channels inactivate fast and the peak current is comparable to that of the P/Q-type Ca^2+^ current in human α-cells). Interestingly, the tight coupling between P/Q-type Ca_v_ channels and exocytosis is preserved in human α-cells, whereas the L-type Ca_v_ channels are involved in Ca^2+^ oscillations. It is possible that the latter contributes to the generation of human α-cell electrical activity. However, it is puzzling that the L-type Ca_v_ channel blocker isradipine only produced a 25% reduction in hypoglycaemia-induced glucagon secretion from human islets and did not abolish glucose sensing in α-cells. This is in stark contrast to the 75% inhibition exerted by the P/Q-type Ca_v_ channel blocker ω-agatoxin VIA, which also rendered glucagon secretion glucose blind. This raised an interesting perspective that glucose metabolism can directly regulate human α-cell exocytosis via effects on the P/Q-type Ca_v_ channels, which was later experimentally demonstrated by the MacDonald group (Dai *et al.* 2022). As such, glucose can control glucagon secretion at a level that is independent of α-cell electrical activity, preventing unwanted spontaneous glucagon release at high glucose (where electrical activity often persists). Apart from the HVA Ca_v_ channels (L-, N-, and P/Q-type), α-cells also express low-voltage-activated T-type Ca_v_ channels, which may function as the pacemaker for AP firing (Rorsman 1988). In addition to Na_v_ and Ca_v_ channels, voltage-gated K^+^ channels (K_v_ channels) also participate in α-cell APs by forming their repolarising phase (downstroke) (Spigelman *et al.* 2010). α-cells possess large K^+^ currents that flow through several types of K_v_ channels. Whereas the mRNA of K_v_2.1, K_v_3.3, K_v_4.1, and Ca^2+^-dependent voltage-sensitive BK channels is detected in mouse α-cells (DiGruccio *et al.* 2016), pharmacological analysis demonstrates that the majority of K^+^ current is mediated by K_v_2.1 and BK channels (Spigelman *et al.* 2010). α-cell K_v_ currents are comprised of two components: a rapid-activating and inactivating component (A-current) that is sensitive to K_v_4.x-blocker heteropodatoxin-2 and a sustained component that can be blocked by stromatoxin (a K_v_2.1/2.2-specific blocker) (Ramracheya *et al.* 2010). The large K^+^ current enables the rapid repolarisation of α-cells, sometimes leading to post-depolarisation hyperpolarisation, essential for reactivating Na_v_ channels and regenerative AP firing. Blocking K_v_ channels leads to increased β-cell electrical activity and insulin secretion by broadening the AP duration (Atwater *et al.* 1979). However, tetraethylammonium (TEA, a broad-spectrum K_v_ channel blocker) inhibits glucagon secretion. This was attributed to TEA-dependent α-cell membrane depolarisation that inactivates Na_v_ and Ca_v_ channels, disabling AP regeneration (Spigelman *et al.* 2010). As such, K_v_ channels are positive regulators of α-cell electrical activity/glucagon secretion.

### Are K_ATP_ channels key to α-cell glucose sensing?

As discussed above, the voltage-sensitive channels are essential apparatus for generating α-cell APs. Due to their electrophysiological properties, particularly the voltage-dependent inactivation of Na_v_ channels, the exact membrane potential of the α-cell is critical for AP firing and glucagon secretion. α-cell membrane potential is determined by its background ionic conductance, formed by several ion channels, including the ATP-sensitive K^+^ channels (K_ATP_ channels).

Like β-cells, α-cells are equipped with K_ATP_ channels, an inwardly rectifying K^+^ channel whose activity is controlled by the intracellular ATP/ADP ratio (Bokvist *et al.* 1999). In β-cells, a low ATP/ADP ratio at low glucose maximally opens the K_ATP_ channels (~2 nS) (Göpel *et al.* 1999), setting the membrane potential close to the K^+^ reversal potential (~–70 mV), where no electrical activity is generated. Interestingly, although molecularly identical to that of β-cells, α-cell basal K_ATP_-channel activity is much lower (~0.1 nS at 1 mM glucose) (Zhang *et al.* 2013). This is possibly because of a high intracellular ATP level in α-cells even when extracellular glucose is low. Indeed, washing out ATP from α-cells rapidly increases their K_ATP_ conductance (Zhang *et al.* 2013). The exact source of the ATP restricting basal K_ATP_-channel activity in α-cells is unclear, but it may be due to high-level glucose transport and metabolism at low glucose levels. α-cells are equipped with GLUT1, a high-affinity glucose transporter (*K*_m_ = 1–2mM) (Heimberg *et al.* 1995), and a low-*K*_m_ sodium–glucose co-transporter SGLT1 (*K*_m_ = 0.5 mM) (Suga *et al.* 2019), which can import glucose at low ambient glucose levels. Moreover, unlike β-cells, α-cells express the high-affinity glycolytic enzyme hexokinase‐1 (HK1, *K*_m_ = ~1 mM) (DiGruccio *et al.* 2016), which phosphorylates glucose to provide substrates for glycolysis. Together, these may explain how α-cells can utilise glucose to generate ATP at low ambient glucose levels.

Interestingly, α-cells remain active and glucagon secretion persists in the complete absence of glucose (Rorsman & Hellman 1988, Gromada *et al.* 1997, Vieira *et al.* 2007). It is unclear how they remain active under glucose deprivation, given AP firing is ATP demanding (Attwell & Laughlin 2001). One possibility is that the creatine/phosphocreatine ATP-buffer system can transfer phosphate to ADP to produce ATP, as it does in β-cells (Krippeit-Drews *et al.* 2003). Moreover, it was proposed that α-cells can generate ATP via fatty acid oxidation (Briant *et al.* 2018). In mice with α-cells lacking CPT1, an enzyme that shuttles fatty acids into the mitochondria, fasting blood glucose and glucagon are reduced (Briant *et al.* 2018). This echoes the observation that reducing lipogenesis in α-cells by knocking out acetyl-CoA-carboxylase 1 dampens glucose sensitivity, an effect linked to an impaired K_ATP_-channel activity (Veprik *et al.* 2022).

The metabolic sensitivity of the K_ATP_ channel makes it a possible fuel sensor of α-cells, similar to its role in β-cells. In both human and mouse islets, increasing extracellular glucose (from 1 to 6 mM) reduces α-cell K_ATP_-channel conductance by ~25% (Bokvist *et al.* 1999, Göpel *et al.* 2000, Zhang *et al.* 2013, Basco *et al.* 2018), an effect exerted by glucokinase (GCK)-dependent glucose metabolism. This depolarises the α-cell membrane to a potential (from ~–55 mV at 1 mM glucose to ~–45 mV at 6 mM glucose) where activatable Na_v_ channels are reduced (from >60% to ~25%) and thus the amplitude of the APs is significantly reduced (Göpel *et al.* 2000, MacDonald *et al.* 2007). The low-amplitude APs can only open a fraction of the exocytosis-related P/Q-type Ca_v_ channels; hence cell exocytosis is greatly reduced (~10% of that at 1 mM glucose). This is consistent with the observation that the K_ATP_-channel inhibitors sulphonylureas can potently inhibit hypoglycaemia-stimulated glucagon secretion.

**Figure 3:**
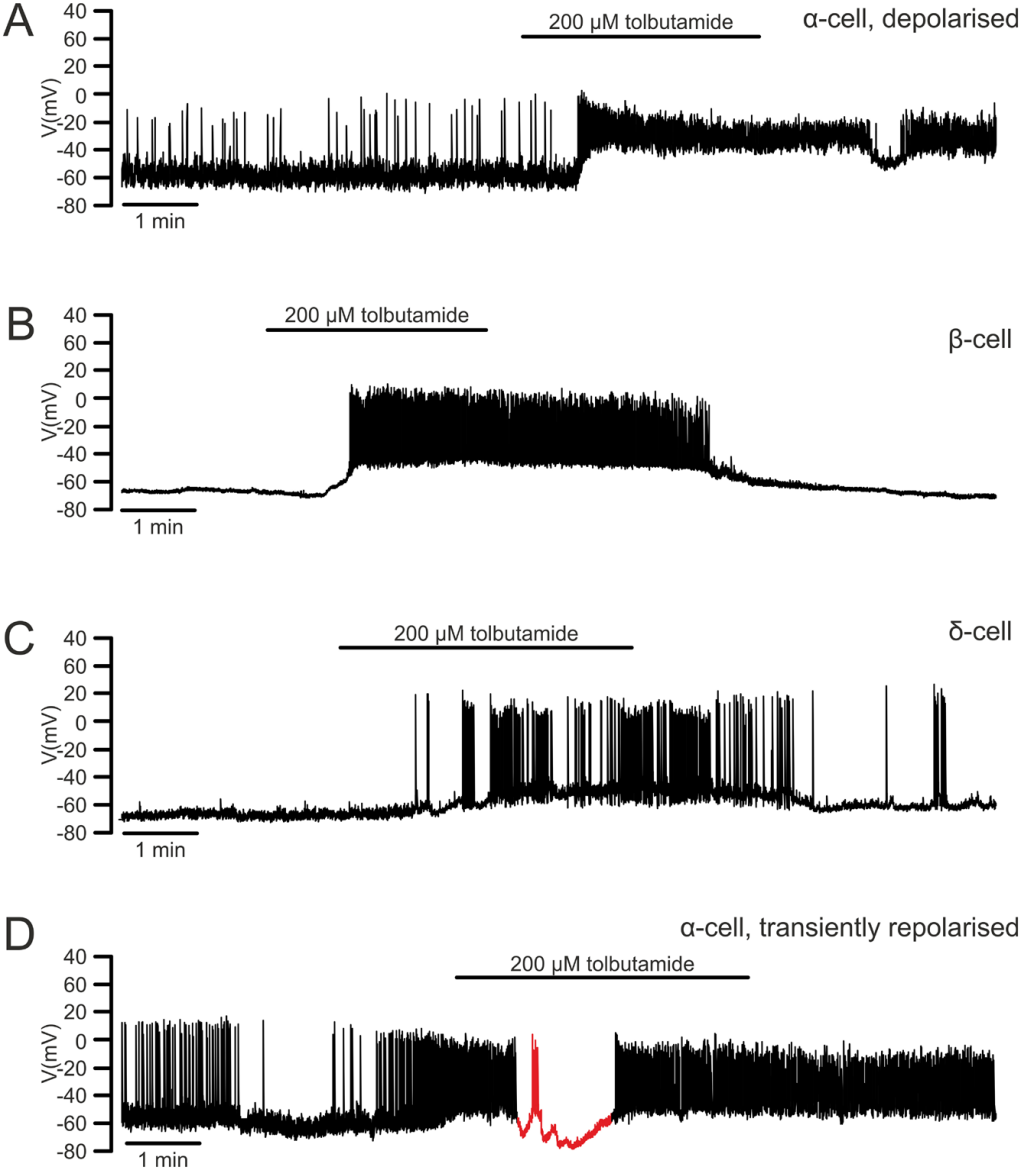
α-, β- and δ-cell membrane potential in response to the sulphonylurea tolbutamide. (A) An example of an α-cell depolarised by 200 μM tolbutamide. (B)–(C). As in A but shows the responses seen in β- and δ-cells. (D) An example of dual effect of tolbutamide on α-cell membrane potential; note the transient repolarisation (red) shortly after the application of tolbutamide.

Interestingly, the effect of sulphonylureas on α-cell electrical activity is not unvarying when tested in intact islets. Tolbutamide, a sulphonylurea, strongly depolarises most α-cells (Fig. 3A), an effect similarly observed in β- and δ-cells (Fig. 3B and C). However, we noticed that, in a small fraction of α-cells, tolbutamide exerted a paradoxical effect on their membrane potential: it induced transient hyperpolarisation (to ~–80 mV) and suppressed AP firing in between depolarisations and continuous electrical activity (Fig. 3D). Whereas we attribute the depolarisation to a direct effect on α-cells, the hyperpolarisation is likely to be due to paracrine effect exerted by stimulated neighbouring δ-cells (Cheng-Xue *et al.* 2013). Indeed, tolbutamide-induced hyperpolarisation is completely absent in dispersed single α-cells (Gromada *et al.* 2004), where paracrine signalling is removed. This mixed effect on α-cell membrane potential was also observed in the presence of high glucose and hyperpolarisation could be reversed by blocking somatostatin receptors (Zhang *et al.* 2013). As such, sulphonylureas could inhibit glucagon secretion through a dual action: they depolarise α-cells to reduce AP and exocytosis and increase intra-islet paracrine tone to further suppress glucagon secretion. The importance of the sulphonylurea-mediated paracrine effect was highlighted by studies from the Gilon group, where a stimulatory effect of K_ATP_-channel blockers on glucagon secretion was observed in somatostatin-deficient mice (Cheng-Xue *et al.* 2013, Singh *et al.* 2021). Clearly, future studies using α-cell-specific K_ATP_-channel deficient mice could help address the precise role of K_ATP_ channels in α-cell intrinsic glucose sensing.

Glucose-induced depolarisation may not only be mediated by K_ATP_-channel closure. It was reported that glucose can be co-transported with Na^+^ through SGLT2 (at a 1:1 ratio) into α-cells (Bonner *et al.* 2015). This transport is theoretically electrogenic and should induce rapid depolarisation to reduce glucagon secretion. Indeed, the SGLT2 blocker dapagliflozin stimulates glucagon secretion at high glucose. However, there is evidence that the Na^+^/glucose co-transport action alone does not produce a depolarisation sufficient to inhibit glucagon secretion. For instance, the non-metabolizable glucose analogue 3-*O*-methyl-d-glucose, which is co-transported with Na^+^ through SGLT2, does not inhibit low-glucose-stimulated glucagon secretion (Cheng-Xue *et al.* 2013).

Apart from the depolarisation hypothesis for glucose-suppressed glucagon secretion, glucose-induced (intrinsic) membrane repolarisation has been observed in several laboratories. It was reported that activation of the two‐pore K^+^ channel TWIK (tandem of P domains in a weak inward rectifying K+ channel)-related acid-sensitive K^+^ channel 1 (TASK1 channel) at high glucose contributed to reduced α-cell excitability. Blockade or genetic ablation of TASK1 depolarised membrane and stimulated AP firing at high glucose (Dadi *et al.* 2015). As such, TASK1 may be an additional α-cell glucose sensor. It was also suggested that α-cell electrical activity at low glucose is maintained by store-operated depolarising Ca^2+^ currents through Orai1 channels (Liu *et al.* 2004). At high glucose, activated sarco-endoplasmic reticulum Ca^2+^-ATPase (SERCA) pumps Ca^2+^ into the ER, inactivating ER‐bound Ca^2+^-sensing stromal interaction molecules (STIMs). This closes Orai1 and the α-cell repolarises, suppressing AP firing and glucagon secretion. However, depleting ER Ca^2+^ using thapsigargin did not abolish glucose sensitivity of glucagon secretion, arguing against a strong involvement of STIM/Orai in α-cell excitability (Gromada *et al.* 2004).

Other mechanisms suggested to mediate glucose-induced α-cell hyperpolarisation are activation of the Na^+^/K^+^ pump (Bode *et al.* 1999) and glucose-induced cell swelling (Davies *et al.* 2007) with subsequent Cl^–^^–^ influx through volume-regulated channels (Best *et al.* 2010). It is worth noting that the Na^+^/K^+^ pump was later proposed to maintain low α-cell membrane potential at low glucose by a CPT1/β-oxidation-dependent mechanism; at high glucose, α-cells switch to glucose metabolism, which produces membrane depolarisation via the closure of the K_ATP_ channels (Briant *et al.* 2018). An interesting Cl^−^ channel in the α-cells is the cAMP-activated cystic fibrosis transmembrane conductance regulator (CFTR). There is still much to learn on this topic, but we and others have detected CFTR on the cell surface of rodent and human α-cells (Edlund *et al.* 2017, Huang *et al.* 2017) and recorded CFTR currents in human α-cells (Edlund *et al.* 2017). RNA sequencing of sorted islets cells did reveal CFTR transcripts in the α-cell fraction but at low levels (Blodgett *et al.* 2015). Although it is still unclear what proportion of the α-cells express CFTR, or at what levels, both mathematical modelling and experimental evidence suggest that when it is present, CFTR exerts a glucagonostatic effect by repolarising α-cells (Edlund *et al.* 2017). Interestingly, Yu *et al.* also recently reported that glucose can directly regulate α-cell intracellular cAMP: low levels of glucose could induce cAMP elevation that is independent of paracrine signalling from insulin or somatostatin (Yu *et al.* 2019). This may have a direct impact on α-cell electrical activity and excoytosis (Omar-Hmeadi *et al.* 2020), effectively regulating glucagon secretion.

Glucagon secretion is not only controlled by glucose but also by other nutrients such as amino acids. Arginine is a strong glucagon secretagogue (Gerich *et al.* 1974). As a cationic amino acid, its transmembrane transport action is electrogenic (via CAT2 which is highly expressed in α-cells (DiGruccio *et al.* 2016)) and normally leads to a large increase in intracellular Ca^2+^ concentration (Le Marchand & Piston 2012). Interestingly, arginine’s stimulatory effect is biphasic (Gerich *et al.* 1974). It is tempting to speculate that the first spike of glucagon secretion is caused by a large but transient membrane depolarisation (due to positive charge influx) and the second phase is due to arginine metabolism (Le Marchand & Piston 2012). Glycine, another amino acid, when bound to its receptor, a ligand-gated Cl^–^ channel, should in principle repolarise α-cells, reducing intracellular Ca^2+^ and glucagon secretion. However, it has been reported that glycine stimulates glucagon secretion and α-cell intracellular Ca^2+^ concentration in human islets (Li *et al.* 2013). It is possible that the intracellular Cl^–^concentration in human α-cells is high and glycine could therefore exert a depolarising effect that is similar to that on human β-cells (Yan-Do *et al.* 2016). Exactly how the amino acids affect α-cell electrical activity requires more detailed electrophysiological analyses. Furthermore, a recent study identified that metabolites, including lactate and pyruvate, robustly inhibit human and mouse α-cell secretion without apparent effect on β- or δ-cells. Lactate entry into α-cells results in K_ATP_-channel activation, membrane hyperpolarisation and reduced [Ca^2+^]_i_ under low glucose conditions (Zaborska *et al.* 2020).

**Figure 4:**
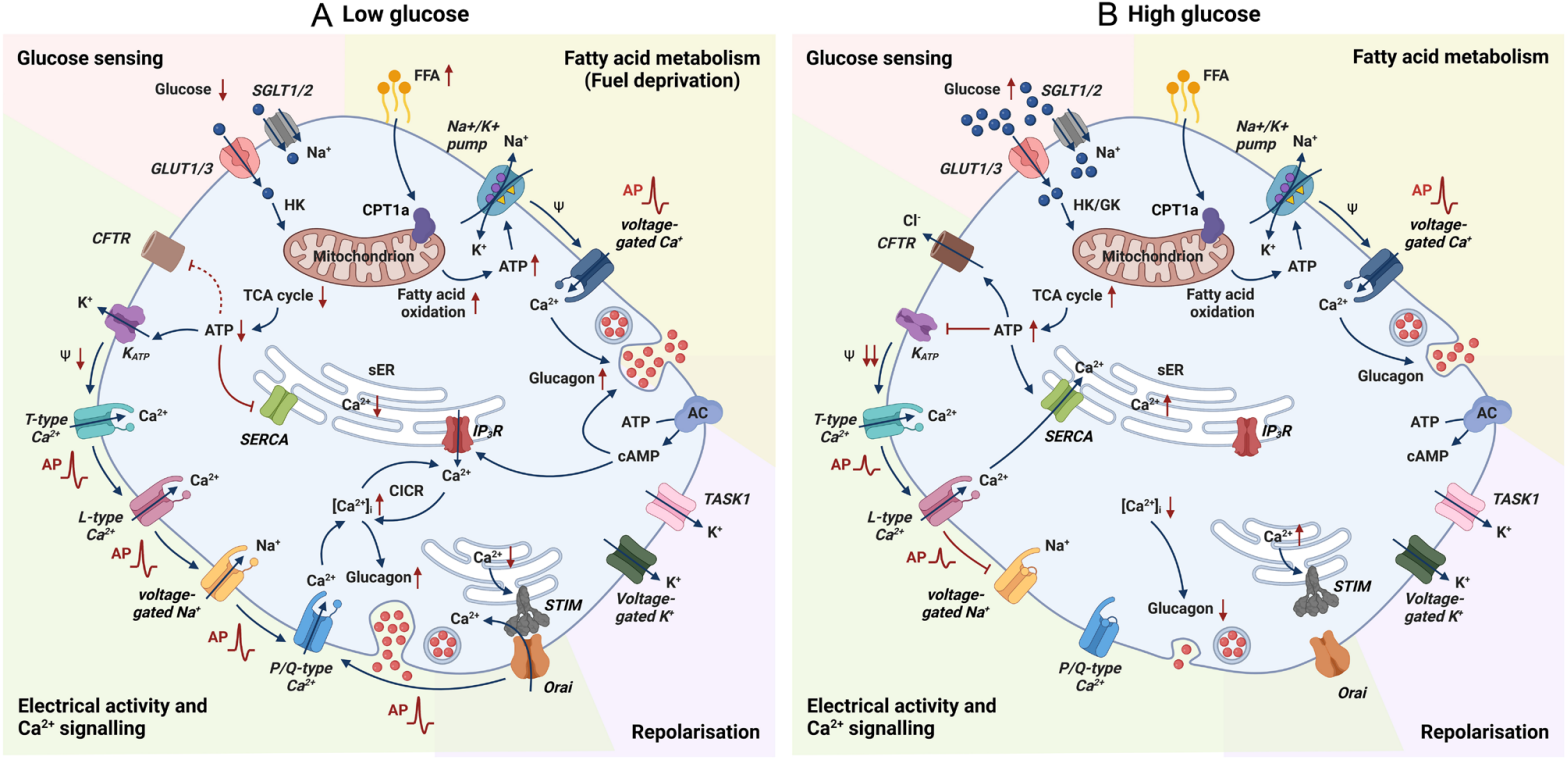
An atlas of ion channel activities in α-cells at (A) low glucose and (B) high glucose. (A) At low glucose, there is a low rate of glucose uptake via glucose transporters (GLUTs) and sodium-glucose linked transporters (SGLTs). Glucose is then metabolised by hexokinase (HK) to modestly increase cytoplasmic ATP/ADP ratio (red-shaded area), leading to partial closure of K_ATP_ channels. This maintains the α-cell membrane potential (*Ψ*) sufficiently depolarised to allow action potential (AP) firing initially driven by T-type Ca^2+^ channels while also preventing voltage-dependent inactivation of the voltage-gated Na^+^ channels. The resulting high-amplitude AP activates P/Q-type Ca^2+^ channels, and the following Ca^2+^ influx amplifies [Ca^2+^]_i_ through potentiation of Ca^2+^ induced Ca^2+^ release (CICR) from the endoplasmic reticulum (ER), further triggering exocytosis of glucagon-containing secretory granules. The relatively low ATP level at low glucose also inhibits sarco/endoplasmic reticulum Ca^2+^-ATPase (SERCA) activity. The subsequent ER Ca^2+^ depletion triggers the activation of store-operated Ca^2+^ current flowing through Orai channels, also contributing to the firing of AP (green-shaded area). Additionally, during the fuel deprivation state when glucose becomes low, fatty acids play a significant role in sustaining basal glucagon secretion. ATP generated by fatty acid oxidation energises the Na^+^–K^+^ pump, keeping the α-cell membrane potential sufficiently repolarised to prevent inactivation of ion channels involved in AP firing (yellow-shaded area). Moreover, two-pore K^+^ channel TWIK-related acid-sensitive K^+^ channel 1 (TASK1) and voltage-dependent K^+^ channels are involved in the repolarisation of α-cells (purple-shaded area). (B) At high glucose, increased glucose transport and associated elevation in ATP/ADP (red-shaded area) trigger complete closure of K_ATP_ channels, strong membrane depolarisation, inactivation of voltage-gated Na^+^ channels, reduced AP amplitude and less Ca^2+^ influx through P/Q-type Ca^2+^ channels. In parallel, ER Ca2+ stores are replenished by ATP-activated SERCA update of [Ca2+]i. This may inactivate Orai-mediated Ca2+ entry into the cytosol, reducing cell excitability. The above two mechanisms together reduce [Ca^2+^]_i,_ culminating in the suppression of glucagon secretion (green-shaded area). When glucose is elevated, cystic fibrosis transmembrane conductance regulator (CFTR) could be activated by intracellular metabolites (i.e. ATP and cAMP). CFTR-mediated Cl- efflux regulates the membrane potential through an intrinsic α-cell effect, also resulting in the inhibition of glucagon secretion. AC, adenylyl cyclase; FFA, free fatty acids; CPT1a, carnitine palmitoyltransferase 1a; GK, glucokinase; IP_3_R, inositol trisphosphate receptor; STIM, stromal interaction molecule. Created with BioRender.com.

Undoubtedly, the nutrient and metabolite control of α-cell electrical activity is complex and involves multiple ion channels and metabolic pathways (Fig. 4A and B). The theories discussed above are not mutually exclusive but exactly how α-cells sense environmental metabolic status and respond accordingly is likely to remain a hot topic of debate for years to come. Are K_ATP_ channels the key to intrinsic glucose sensing in α-cells? Clearly, their strong tonic inhibition sets a high membrane resistance of the cells at nearly all glucose concentrations. This enables the α-cell electrical activity/membrane potential and function to be significantly altered by minute changes to the activity of either the K_ATP_ channels or other ion channels.

## Paracrine and neuronal control of α-cells

**Figure 5:**
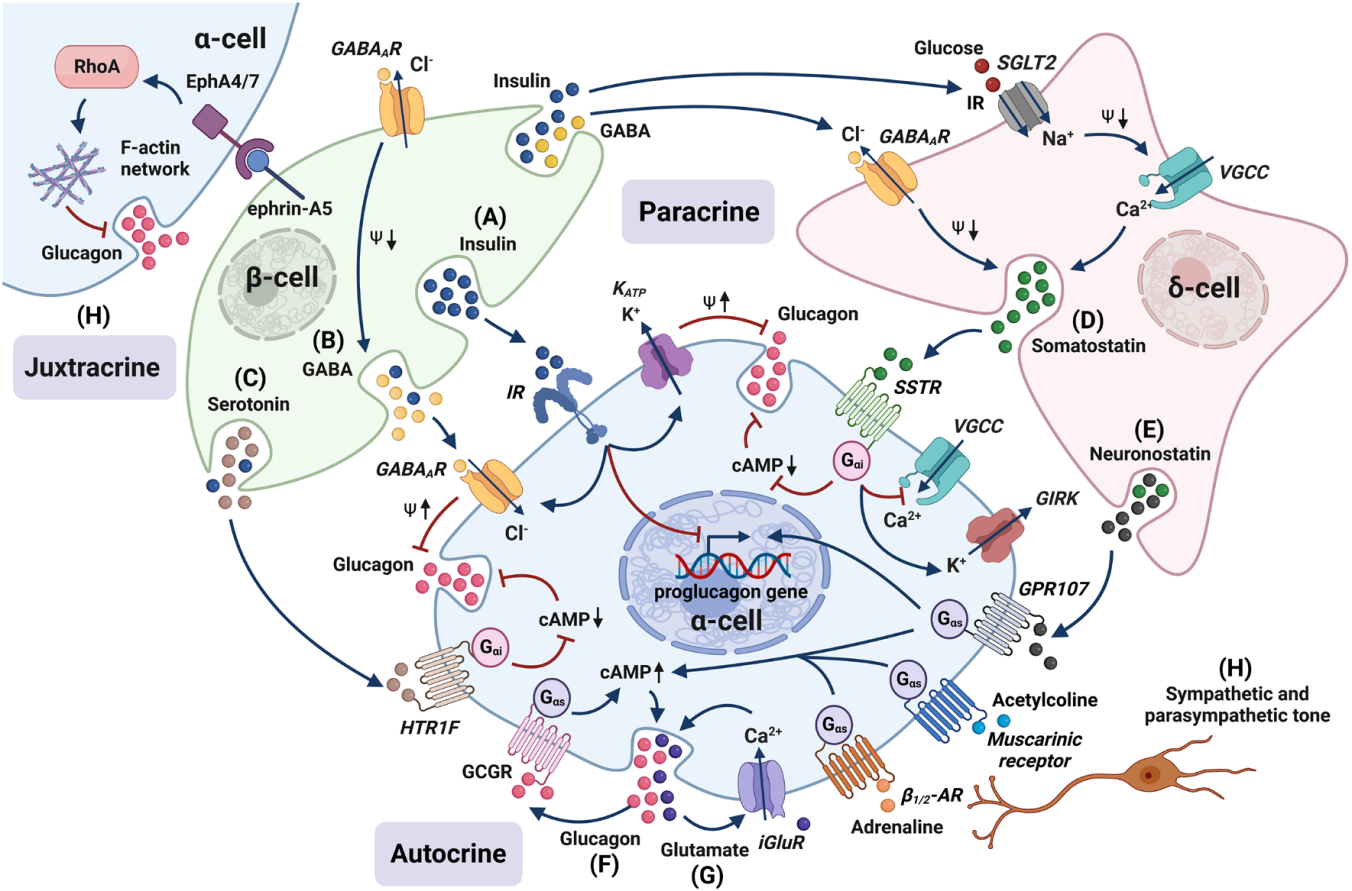
Paracrine, autocrine, and juxtracrine regulation of α-cell. In addition to glucose, numerous paracrine, autocrine, hormonal and nervous signals fine-tune glucose secretion under different physiological conditions. (A) Upon binding to insulin receptor (IR), insulin inhibits glucagon secretion via increasing K_ATP_-channel activity, potentiating γ-aminobutyric acid (GABA) signalling by promoting the localisation of type A GABA receptors (GABA_A_) to the plasma membrane, and inducing reduction in α-cell proglucagon gene transcription. Insulin could also indirectly decrease glucagon release by stimulation of intra-islet somatostatin secretion via δ-cell sodium-glucose linked transporter 2 (SGLT2). (B) While GABA on one hand has a direct inhibitory effect on glucagon secretion through GABA_A_ receptor activation with a resulting hyperpolarising Cl^–^ current in α-cells, on the other hand it stimulates insulin and somatostatin release which further leads to α-cell inhibition. (C) Serotonin is co-secreted with insulin and inhibits glucagon release via activation of G_αi_-coupled serotonin receptor 1F (HTR1F) resulting in decreased cAMP levels. (D) The downstream signalling of somatostatin by activation of somatostatin receptor (SSTR) on α-cells involves activation of G_αi_ protein leading to decreased cytoplasmic levels of cAMP; hyperpolarisation and inhibition of action potential firing via G protein-coupled inwardly rectifying K^+^ (GIRK) channels; and inhibition of Ca^2+^ influx through voltage-gated P/Q-type Ca^2+^ channels. (E) Neuronostatin-mediated increase in glucagon secretion by binding to GPR107, triggering cAMP-independent protein kinase A (PKA) phosphorylation and proglucagon mRNA accumulation in α-cells. (F) Glucagon released by α-cell could stimulate its own secretion as an autocrine regulator by binding to glucagon receptor (GCGR) and promoting downstream cAMP generation. (G) Glutamate is also a positive autocrine signal for glucagon release. By acting on ionotropic glutamate receptors (iGluRs) of the AMPA/kainate type, glutamate enhances glucagon release via membrane depolarisation and opening of voltage-gated Ca^2+^ channels (VGCC). (H) Sympathetic and parasympathetic tones also modulate α-cell glucagon secretion. (I) α- and β-cell juxtracrine signalling is mediated by EphA/ephrin-A pathway and downstream RhoA activity. β_1/2_-AR, β_1/2_ adrenergic receptor. Created with BioRender.com.

The theories regarding how glucose controls glucagon secretion are not limited to intrinsic mechanisms. Many have proposed that α-cell glucagon secretion is controlled by neighbouring cells and/or neuronal regulation (Fig. 5).

## Control by β-cells

### Insulin

Insulin has long been considered the regulator for glucagon secretion, and insulin receptors as well as proteins involved in insulin signalling are highly expressed in α-cells (DiGruccio *et al.* 2016). Indeed, knocking-out insulin receptors in α-cells led to hyperglucagonemia and mild glucose intolerance (Kawamori *et al.* 2009). Upon binding to its receptor, insulin activates the phosphatidylinositol 3 kinase/Akt-dependent pathway (Kaneko *et al.* 1999), which reduces the K_ATP_-channel ATP sensitivity in α-cells, dampening their excitability (Leung *et al.* 2006). Furthermore, insulin was reported to induce FoxO1 nuclear exclusion, subsequently reducing proglucagon gene transcription (McKinnon *et al.* 2006) with an impact on long-term glucagon maintenance.

### γ-aminobutyric acid

γ-aminobutyric acid (GABA) is present in islets and has been located to insulin vesicles (Braun *et al.* 2007) and synaptic like microvesicles (Thomas-Reetz *et al.* 1993) in β-cells, from where it is released in a glucose- and Ca^2+^-dependent manner (Braun *et al.* 2010, Braun *et al.* 2004). More recently, it was reported that GABA is also present in the β-cell cytosol and can be secreted via non-vesicular release mediated by volume-regulated anion channels (Menegaz *et al.* 2019).

In human α-cells, mRNA transcripts for both ionotropic GABA_A_ receptors and metabotropic GABA_B_ receptors are detectable (Blodgett *et al.* 2015). However, GABA_B_ receptors probably play a limited role since the GABA_B_ receptor antagonist CGP555845 does not affect glucagon secretion in human islets (Taneera *et al.* 2012). In contrast, ample evidence suggests that GABA_A_ receptor activation in human and rodent islets reduces glucagon secretion (Rorsman *et al.* 1989, Wendt *et al.* 2004, Taneera *et al.* 2012). Interestingly, we found that insulin potentiates GABA signalling by promoting membrane localisation of GABA_A_ receptors, linking the two β-cell-derived factors in combined paracrine control of glucagon secretion (Xu *et al.* 2006). GABA_A_ receptors are ligand-gated Cl^–^ channels and, in most cases, their activation hyperpolarises the cell, reducing AP generation. This is somewhat different in human islets. Immunostaining shows GABA_A_ receptor expression in human α-cells (Taneera *et al.* 2012), and functional GABA_A_ currents were recorded in the same cells. However, Braun *et al.* found GABA_A_ currents from human α-cell to be relatively small and only detectable in a subset of α-cells (Braun *et al.* 2010); instead, large GABA_A_ currents from both β- and δ-cells were detected. Surprisingly, GABA_A_ receptor activation in the latter cells is depolarising (due to their high intracellular Cl^–^ concentration) and hence stimulatory for insulin and somatostatin secretion. This opens up two potential pathways for GABA to affect α-cells. GABA could have a direct inhibitory effect on glucagon secretion via GABA_A_ receptor activation with a resulting hyperpolarising Cl^–^ current in the α-cells (in the case of low intracellular Cl^–^ concentration); and/or GABA stimulates insulin and somatostatin secretion to inhibit glucagon secretion. A better understanding of how/whether these two mechanisms synergistically function requires more detailed investigations.

Interestingly, long-term exposure to high glucose (>1 h) promotes β-cell GABA catabolism by shunting GABA into the citric acid cycle via the ‘GABA shunt’ (Wang *et al.* 2006, Pizarro-Delgado *et al.* 2010). This reduces both the intracellular content and release of GABA when stimulated acutely with glucose. Therefore, prolonged hyperglycaemia can also affect the GABA-mediated β-cell paracrine control of glucagon secretion.

### Zinc and serotonin

Zn^2+^ crystallises with insulin in large dense-core vesicles and is co-released with insulin from β-cells (Hardy *et al.* 2011). Zn^2+^ was shown to inhibit pyruvate-stimulated glucagon secretion in perfused rat pancreas (Ishihara *et al.* 2003), an effect that was confirmed by static secretion and electrophysiological experiments in purified rat α-cells (Franklin *et al.* 2005). Later studies, conducted in a hypoglycaemic state, found that switching off either free Zn^2+^ or Zn^2+^ bound to insulin, rather than insulin itself, represents the ‘switch-off’ signal from β-cells to α-cells that initiates glucagon secretion (Zhou *et al.* 2007). The mechanism by which Zn^2+^ reduces glucagon secretion involves the opening of α-cell K_ATP_ channels that dampens α-cell excitability and restricts the opening of Ca^2+^ channels (Franklin *et al.* 2005, Slucca *et al.* 2010). However, possibly due to different experimental settings and species differences, several groups have reported that Zn^2+^ does not suppress glucagon secretion or intracellular Ca^2+^ in human islets and mouse α-cells (Ravier & Rutter 2005, Quoix *et al.* 2009, Ramracheya *et al.* 2010). Furthermore, whole-body ZnT8 (Zn^2+^ transporter) deletion has no effect on glucagon secretion (Nicolson *et al.* 2009).

Serotonin is also co-secreted with insulin and activates G_αi_-coupled serotonin receptor 1F (HTR1F) on neighbouring α-cells, resulting in decreased cAMP levels and suppression of glucagon secretion (Almaca *et al.* 2016). This study also suggested that reduced serotonergic control of α-cells can be a contributing factor for glucagon dysregulation in diabetes.

### Control by δ-cells

In mouse islets, somatostatin-releasing δ-cells are localised in islet periphery and close to α-cells, while in humans they are scattered throughout the islets (Brereton *et al.* 2015). δ-cells exhibit a neuron-like morphology with processes that can reach several cell layers (Arrojo *et al.* 2019). This enables the low-population δ-cells (~10% of the islet cells) to exert islet-wide paracrine regulation. Somatostatin is a powerful inhibitor of glucagon (Xu *et al.* 2020), and its effect on α-cells is primarily mediated by somatostatin receptor 2 (SSTR2) both in mouse and in human islets (Gromada *et al.* 2001, Kailey *et al.* 2012). SSTR2 is a G_αi_-coupled receptor, and its activation inhibits α-cells via (i) decreasing adenylyl cyclase activity and cytoplasmic cAMP levels (Elliott *et al.* 2015); (ii) activating G protein-coupled inwardly rectifying K^+^ channels to reduce cellular excitability (Kailey *et al.* 2012); and (iii) inactivating Ca_v_ channels. In mice, glucagon release is increased across the full range of physiological glucose levels when somatostatin is knocked out (Cheng-Xue *et al.* 2013) or SSTR2 is blocked (Lai *et al.* 2018), suggesting tonic δ-cell inhibition of α-cells. It is well established that the paracrine action of somatostatin contributes to the glucagonostatic effect of high glucose, although the functions/mechanisms of somatostatin release at low glucose remain to be understood.

δ-cells also release neuronostatin, a peptide produced from pro-somatostatin (Samson *et al.* 2008). Neuronostatin was shown to increase glucagon secretion by binding to GPR107, resulting in cAMP-independent PKA phosphorylation and proglucagon mRNA accumulation in α-cells (Elrick *et al.* 2016). However, the physiological importance of neuronostatin in the control of glucose homeostasis remains to be determined.

### α-cell autocrine and juxtracrine control

In addition to intra-islet paracrine signalling, α-cell autocrine control of glucagon secretion has also been reported. It was found that glucagon could stimulate α-cell exocytosis by binding to glucagon receptors (GCGR), promoting cAMP generation (Ma *et al.* 2005). Glucagon also regulates its own synthesis in α-cells by signalling through GCGR, protein kinase C (PKC), and PKA, suggesting a long-term autocrine effect on hormone synthesis (Leibiger *et al.* 2012). α-cells also produce and release glutamate, an excitatory neurotransmitter. Acting on ionotropic glutamate receptors (iGluRs) of the AMPA/kainate type, glutamate enhances glucagon release via membrane depolarisation and opening of Ca_v_ channels (Cabrera *et al.* 2008).

Juxtracrine signalling in α-cells comes primarily through direct contact with β-cells and work synergistically with paracrine and autocrine control of glucagon release (Hughes *et al.* 2018). In particular, the EphA/ephrin-A pathway has been shown to regulate glucagon secretion via α-cell EphA4 interactions with ephrin-A5 expressed on the surface of β-cells (Hutchens & Piston 2015). Stimulation of EphA4 suppresses glucagon secretion by modulating the activity of RhoA, a signalling hub that affects Ca^2+^ signalling, cortical F-actin density and exocytosis in α-cells (Ng *et al.* 2022).

### Central nervous system control of α-cells

The central nervous system works in tandem with islets to maintain glucose homeostasis via direct autonomic innervation, indirect neuroendocrine mechanisms and glucose sensing to modulate glucose counter-regulation (Faber *et al.* 2020).

### The autonomic tone on α-cells

Adrenaline and noradrenaline are released from both islet sympathetic innervation and adrenal medulla. Stress, including hypoglycaemia, triggers the release of these mediators. In human and rodent islets, adrenaline directly stimulates glucagon secretion via activation of β-adrenergic receptors. Possible mechanistic routes are mobilisation of Ca^2+^ from lysosomal acidic stores and ER (Hamilton *et al.* 2018) and increasing cAMP to activate Epac2 (De Marinis *et al.* 2010). Interestingly, it was reported that sympathetic innervation in human islets is restricted to blood vessels (Rodriguez-Diaz *et al.* 2011*a*), questioning whether neuronal modulation directly controls α-cells in human. This is different in type 1 diabetes (T1D) though, where a direct sympathetic innervation of α-cells was demonstrated (Campbell-Thompson *et al.* 2021).

Regarding parasympathetic control of glucagon secretion, acetylcholine stimulates glucagon secretion in rodent islets through binding to muscarinic receptors (reviewed in (Ahrén 2000)). Human α-cells, on the other hand, do not respond to acetylcholine (Molina *et al.* 2014), although they are suggested to be an important source of acetylcholine regulating other islet cells (Rodriguez-Diaz *et al.* 2011*b*).

### The brain as glucose sensor for counter-regulation to hypoglycaemia

Hypoglycaemia-associated autonomic failure (HAAF) is a phenomenon first described by Simon Heller and Philip Cryer, in which insulin-induced hypoglycaemia (IHH) leads to reduced α-cell and sympathetic responses, creating a vicious cycle by increasing the susceptibility to future hypoglycaemic episodes (Heller & Cryer 1991). HAAF is of particular concern in people living with T1D, and a role for brain glucose-sensing neurons has been identified in its mechanism (Cryer 2006).

The glucose-excited and glucose-inhibited neurons in ventromedial nucleus of the hypothalamus (VMH) work in concert to stimulate or inhibit glucose counter-regulation (Sherwin 2008). Previous hypoglycaemia impairs VMH glucose sensing by multiple adaptations, including reduced VMH K_ATP_-channel activity (McCrimmon *et al.* 2005), increased activation of inhibitory neuronal circuits (VMH GABA (Chan *et al.* 2008) or urocortin 3 input (Flanagan *et al.* 2003)), and suppressed VMH AMP kinase activity (Alquier *et al.* 2007). This could lead to insufficient glucagon and adrenaline release during hypoglycaemia. It is worth noticing that these responses are reversible from both clinical and preclinical perspectives; thus, therapies targeting these mechanisms have the potential to restore normal counter-regulation in people living with T1D.

## α-cells in diabetes and targeted anti-diabetic treatments

**Figure 6:**
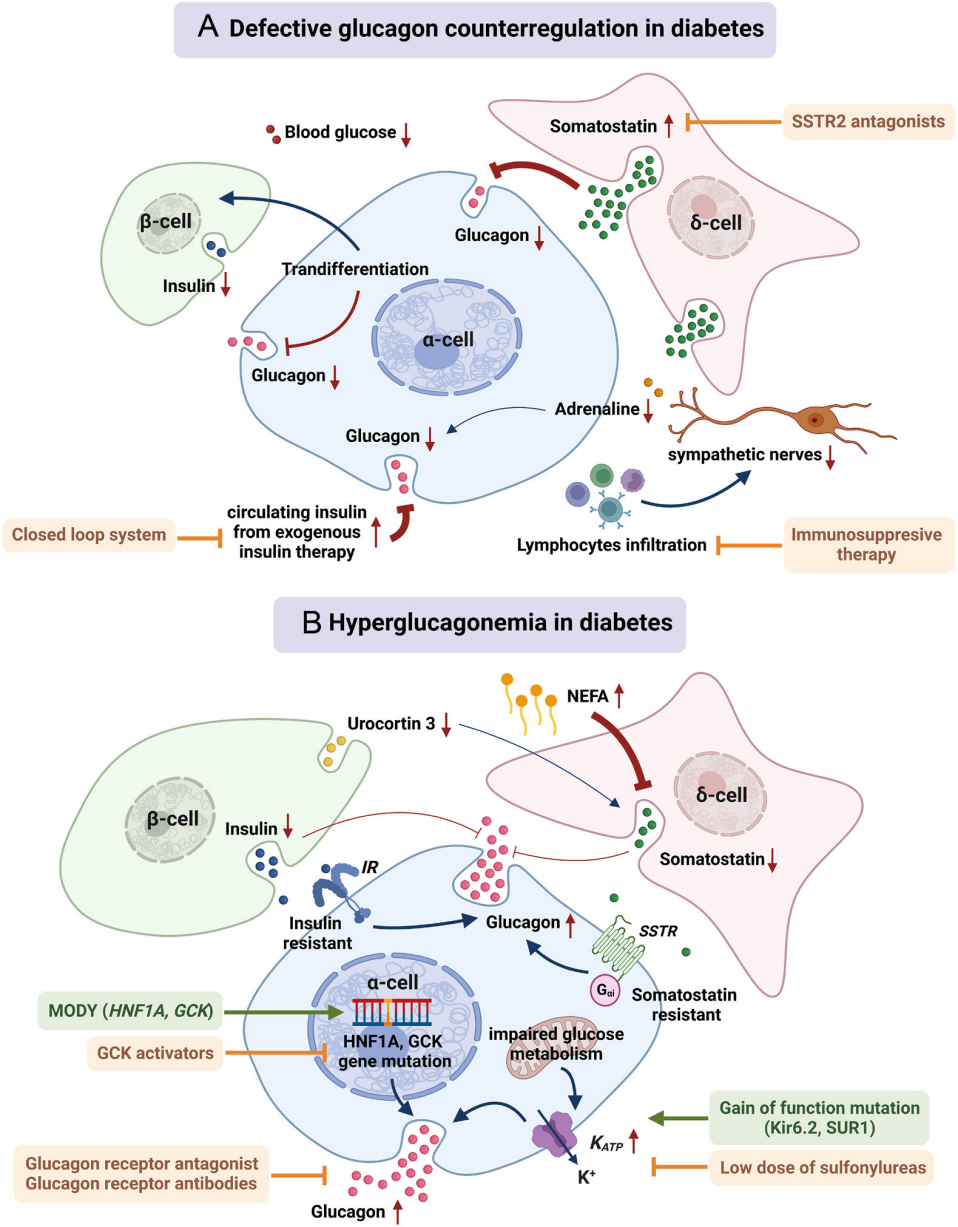
α-cell defects in diabetes and targeted anti-diabetic treatments. Therapeutic strategies are highlighted in orange/brown, while gene defects associated with inappropriate glucagon secretion are highlighted in green. (A) Mechanisms of defective glucagon counter-regulation in diabetes. Inadequate glucagon secretion of α-cell at low glucose could be attributed to intensive insulin regimen triggering strong insulin inhibitory effect on α-cell during hypoglycaemia, excessive somatostatin secretion, impairment of sympathetic tone-mediated glucagon secretion, and α-cell loss of identity by transdifferentiation into β-cells. The abovementioned mechanisms could be counteracted by targeting δ-cell tonic inhibition with somatostatin receptor 2 (SSTR2) antagonist, immunosuppressive therapy inhibiting lymphocytic activation and subsequent islet neuropathy, or the use of closed-loop system automatically modulating insulin pump dose according to continuous glucose monitoring. (B) Mechanisms of hyperglucagonemia in diabetes. α-cell intrinsic defects such as gain-of-function mutation of K_ATP_ channel in neonatal diabetes, *increased K_ATP_channel activity* as a consequence of perturbed α-cell metabolism in type 2 diabetes (T2D), and *HNF1A* and *GCK* mutation in maturity-onset diabetes of the young (MODY) could trigger inadequate glucose-induced suppression of glucagon. Low dose of* K_ATP_* channel blocker sulfonylureas, glucokinase (GCK) activators, and glucagon receptor antagonists are beneficial to counteract the hyperglucagonemia in this group of people living with diabetes. Paracrine defects can also lead to hyperglucagonemia through α-cell insensitivity to paracrine inhibition of insulin and somatostatin. β-cell loss at late stage of diabetes resulting in reduced insulin and urocortin 3-potentiated somatostatin secretion could cause subsequent hyperglucagonemia. Moreover, raised non-esterified fatty acid (NEFA) level in T2D can also inhibit somatostatin release. Created with BioRender.com.

People living with diabetes often present three types of defects in glucagon secretion: (i) impaired glucagon counter-regulation in response to hypoglycaemia, which is frequently seen in T1D (Cryer 2012); (ii) fasting hyperglucagonemia; and (iii) postprandial hyperglucagonemia, which often exacerbates hyperglycaemia in T2D (Shah *et al.* 2000, Dunning & Gerich 2007). Agents targeting glucagon signalling (glucagon receptor antagonists) can effectively attenuate hyperglycaemia in animal models of diabetes (Okamoto *et al.* 2017) and people living with diabetes (Pettus *et al.* 2018). Overall, the current data support the provocative glucagonocentric hypothesis proposed by Unger *et al.* that glucagon excess, rather insulin deficiency, is the *sine qua non* of diabetes (Unger & Cherrington 2012). However, the glucagonocentric hypothesis was later challenged by the observation that, in a streptozotocin (STZ) model of diabetes, α-cell ablation did not relieve the diabetic phenotype, although it is worth noting that removal of α-cells improved glucose tolerance in STZ-treated animals (Steenberg *et al.* 2016). Proposed mechanisms by which glucagon becomes defective in diabetes are illustrated in Fig. 6.

## Mechanisms of defective glucagon counter-regulation

Hypoglycaemia, a common complication of T1D, is partly attributable to inadequate glucagon secretion at low glucose. From the perspective of insulin’s inhibitory action on glucagon, intensive insulin treatment in T1D can cause high circulating insulin during hypoglycaemia, which could inhibit α-cell activity to disable the glucagon response (Raju & Cryer 2005). For δ-cell-related defects, it has been shown that excessive somatostatin secretion contributes to glucagon failure in hypoglycaemia (Yue *et al.* 2013), while SSTR2 antagonists restore hypoglycaemia-stimulated glucagon release, preventing hypoglycaemia in diabetic animals (Karimian *et al.* 2013). However, before translating into clinical application, the safety of SSTR2 antagonists needs to be carefully assessed since SSTR2 is widely expressed in the body (stomach, adrenal medulla, cerebral cortex, hypothalamus, and pituitary gland) (Taleb & Rabasa-Lhoret 2016).

The autonomic nervous system also contributes to glucagon response in IIH by modulating sympathetic and parasympathetic tones (Taborsky & Mundinger 2012). It was found that during the onset of T1D, the majority of islet sympathetic nerves are lost (termed as early sympathetic islet neuropathy) due to lymphocytic infiltration/activation. This results in defective sympathetically mediated glucagon secretion, aggravating IIH (Mundinger & Taborsky 2016). Thus, early-stage immunosuppressive therapy could potentially be beneficial for preventing this neuropathy.

The defective glucagon secretion could also be attributable to the loss of α-cell identity by adopting β-cell features when β-cells are depleted in diabetes (Bru-Tari *et al.* 2019, Furuyama *et al.* 2019). It is possible that this change in identity (α-cell to β-cell) could invert glucose-dependent glucagon secretory pattern if glucagon remains to be produced in transdifferentiated cells. It will be interesting to test to what extent this can affect glycaemic control in animal models and people living with diabetes.

## Mechanisms of hyperglucagonemia

### α-cell-intrinsic defects

*In situ* α-cell electrophysiology revealed that hyperglucagonemia is related to increased AP amplitude and firing frequency, higher Na_v_ current density, and reduced K_v_ current density in a STZ-induced diabetes model (Huang *et al.* 2013). These findings suggest that there is an intrinsic mechanism of glucagon dysregulation.

K_ATP_ channels are involved in the intrinsic glucose-sensing mechanism of α-cells. Gain-of-function mutations in the genes encoding the pore-forming (Kir6.2 and *KCNJ11*) and regulatory (SUR1, *ABCC8*) subunits of the K_ATP_-channel cause neonatal diabetes due to loss of β-cell glucose sensing (Gloyn *et al.* 2004). A common variant (E23K; rs5219) in* KCNJ11* is associated with enhanced T2D risk (Gloyn *et al.* 2003) due to increased K_ATP_-channel activity (Schwanstecher *et al.* 2002) and impaired glucose-induced suppression of glucagon secretion *in vivo* (Tschritter *et al.* 2002). We have suggested that, in T2D, dysregulation of glucagon secretion may be associated with slightly increased K_ATP_-channel activity in α-cells, possibly as a consequence of impaired glucose metabolism (Zhang *et al.* 2013). Indeed, low concentration of K_ATP_-channel blocker tolbutamide restores normal glucose regulation of glucagon release in metabolically compromised and T2D islets. This was then confirmed in clinical trials, where low-dose sulfonylureas (0.3 mg/day glibenclamide) was found to be useful in reducing fasting hyperglucagonemia in people living with T2D (Spiliotis *et al.* 2022).

Maturity-onset diabetes of the young (MODY) is an inherited autosomal dominant condition, most commonly caused by mutations in *HNF1A* (MODY 3) and *GCK* (MODY 2). Although its link to insulin secretory defects has been well investigated, less is known about α-cell pathophysiology in MODY patients. *HNF1A* can control glucagon secretion in α-cells through modulation of SGLT1, and Hnf1a^–/–^ mice showed higher fasting glucagon levels and exhibited inadequate suppression of glucagon after glucose challenge (Sato *et al.* 2020, Saponaro *et al.* 2022). In *HNF1A*-MODY patients, low-dose gliclazide, a sulphonylurea, was found to improve hyperglucagonemia after a glucose challenge (Saponaro *et al.* 2022). However, whether long-term treatment with gliclazide affects α-cell function and the mechanism underlying the treatment response needs further investigation. α-cell GCK plays a central metabolic role in the suppression of glucagon secretion at euglycaemic and hyperglycaemic levels (Basco *et al.* 2018). In GCK-MODY patients, the threshold for glucose to suppress glucagon is higher than that in people without diabetes (Guenat *et al.* 2000). Thus, GCK activators could potentially normalise glucagon secretion by tuning glycolysis and α-cell K_ATP_-channel activity (Nakamura & Terauchi 2015).

Chronic hyperglycaemia was also reported to induce α-cell dysregulation. Mechanistically, impaired ATP production in α-cells is triggered by increased Na^+^ uptake through SGLTs, intracellular and mitochondrial acidification, and protein succination due to reduced fumarase activity (Knudsen *et al.* 2019). This defect can be corrected by low concentrations of tolbutamide and prevented by SGLT inhibitors.

### Paracrine defects

Considering that insulin inhibits glucagon release, β-cell loss and defects in intra-islet insulin-signalling pathway may contribute to the development of diabetic hyperglucagonemia. In T2D, the defective paracrine role of β-cells is supported by the loss of the inverse relationship between pulsatile insulin and glucagon secretion (Menge *et al.* 2011). However, the moderate insulin secretory defects at the initial stage of T2D does not fully support the idea that post-prandial hyperglucagonemia is only due to β-cell dysfunction. It is possible that α-cells develop insulin resistance, including blunted insulin-stimulated Akt phosphorylation, during chronic exposure to high glucose and insulin (Tsuchiyama *et al.* 2007).

Defective glucagon regulation can be caused by impaired paracrine control from δ-cells. It was reported that human T2D islet α-cells develop somatostatin resistance (Omar-Hmeadi *et al.* 2020). This could be responsible for the post-prandial hyperglucagonemia in T2D. In addition, long-term exposure to non-esterified fatty acid (NEFA, often elevated in T2D) reduces glucose-stimulated somatostatin secretion and correspondingly induces a 50% increase in glucagon release (Collins *et al.* 2008). Furthermore, in diabetes, β-cell urocortin-3, a stimulant of δ-cell secretion (van der Meulen *et al.* 2015), is greatly depleted and therefore could be associated with insufficient somatostatin secretion and hyperglucagonemia.

## Conclusion

As the islet cells that produce glucagon, a principal counter-regulatory hormone, the α-cells play a vital role in the prevention of hypoglycaemia and the maintenance of systemic glucose homeostasis. However, although decades (a century if you count from the discovery of glucagon) of research have greatly developed our understanding of the cell, the exact regulatory mechanism(s) of the α-cells remain(s) enigmatic. The α-cell electrophysiology, as part of its physiology, is fascinating and has been the topic of studies into the fundamental aspects of how α-cells function, secrete glucagon, and sense glucose.

The role of glucagon dysregulation in diabetes is recognised, and glucagon receptor antagonists are already emerging as a promising new class of anti-diabetic drugs. It can be predicted that with the α-cells in the limelight of islet research, endeavours for understanding the pathophysiology of glucagon dysregulation will lead to the development of therapies that can restore normal α-cell function in diabetes. This will ultimately offer better diabetes care, particularly in the aspect of hypoglycaemia prevention, and will greatly improve the quality of life of people living with diabetes.

## Declaration of interest

The authors declare that there is no conflict of interest.

## Funding

This work has been supported by a Diabetes UK RD Lawrence Fellowship (14/0005128) (QZ) and an EFSD New Targets for Diabetes or Obesity-related Metabolic Diseases Programme (QZ), the Swedish Foundation for Strategic Research (IRC-LUDC; AW), the Swedish Research Council (SFO-EXODIAB; AW), the Albert Påhlsson Foundation (AW), and National Natural Science Foundation of China (82200887) (RG).

## References

[bib1] Ahrén B 2000 Autonomic regulation of islet hormone secretion – implications for health and disease. Diabetologia 43 393–410. (10.1007/s001250051322) 10819232

[bib2] Almaca J Molina J Menegaz D Pronin AN Tamayo A Slepak V Berggren PO & Caicedo A 2016 Human beta cells produce and release serotonin to inhibit glucagon secretion from alpha cells. Cell Reports 17 3281–3291. (10.1016/j.celrep.2016.11.072) 28009296 PMC5217294

[bib3] Alquier T Kawashima J Tsuji Y & Kahn BB 2007 Role of hypothalamic adenosine 5'-monophosphate-activated protein kinase in the impaired counterregulatory response induced by repetitive neuroglucopenia. Endocrinology 148 1367–1375. (10.1210/en.2006-1039) 17185376

[bib4] Arrojo E Drigo R Jacob S Garcia-Prieto CF Zheng X Fukuda M Nhu HTT Stelmashenko O Pecanha FLM Rodriguez-Diaz R Bushong E, 2019 Structural basis for delta cell paracrine regulation in pancreatic islets. Nature Communications 10 3700. (10.1038/s41467-019-11517-x) PMC6697679 31420552

[bib5] Attwell D & Laughlin SB 2001 An energy budget for signaling in the grey matter of the brain. Journal of Cerebral Blood Flow and Metabolism 21 1133–1145. (10.1097/00004647-200110000-00001) 11598490

[bib6] Atwater I Ribalet B & Rojas E 1979 Mouse pancreatic beta-cells: tetraethylammonium blockage of the potassium permeability increase induced by depolarization. Journal of Physiology 288 561–574. 381635 PMC1281444

[bib7] Barg S Galvanovskis J Göpel SO Rorsman P & Eliasson L 2000 Tight coupling between electrical activity and exocytosis in mouse glucagon-secreting alpha-cells. Diabetes 49 1500–1510. (10.2337/diabetes.49.9.1500) 10969834

[bib8] Basco D Zhang Q Salehi A Tarasov A Dolci W Herrera P Spiliotis I Berney X Tarussio D & Rorsman P 2018 α-cell glucokinase suppresses glucose-regulated glucagon secretion. Nature Communications 9 1–9. (10.1038/s41467-018-03034-0) PMC5803227 29416045

[bib9] Baum J Simons BE Unger RH & Madison LL 1962 Localization of glucagon in the alpha cells in the pancreatic islet by immunofluorescent technics. Diabetes 11 371–374. 13966974

[bib10] Best L Brown PD Sener A & Malaisse WJ 2010 Electrical activity in pancreatic islet cells: the VRAC hypothesis. Islets 2 59–64. (10.4161/isl.2.2.11171) 21099297

[bib11] Blodgett DM Nowosielska A Afik S Pechhold S Cura AJ Kennedy NJ Kim S Kucukural A Davis RJ Kent SC, 2015 Novel observations from next-generation RNA sequencing of highly purified human adult and fetal islet cell subsets. Diabetes 64 3172–3181. (10.2337/db15-0039) 25931473 PMC4542439

[bib12] Bode HP Weber S Fehmann HC & Göke B 1999 A nutrient-regulated cytosolic calcium oscillator in endocrine pancreatic glucagon-secreting cells. Pflugers Archiv 437 324–334. (10.1007/s004240050786) 9914388

[bib13] Bokvist K Olsen HL Høy M Gotfredsen CF Holmes WF Buschard K Rorsman P & Gromada J 1999 Characterisation of sulphonylurea and ATP-regulated K^+^ channels in rat pancreatic A-cells. Pflügers Archiv 438 428–436. (10.1007/s004249900076) 10519134

[bib14] Bonner C Kerr-Conte J Gmyr V Queniat G Moerman E Thevenet J Beaucamps C Delalleau N Popescu I Malaisse WJ, 2015 Inhibition of the glucose transporter SGLT2 with dapagliflozin in pancreatic alpha cells triggers glucagon secretion. Nature Medicine 21 512–517. (10.1038/nm.3828) 25894829

[bib15] Braun M Ramracheya R Bengtsson M Clark A Walker JN Johnson PR & Rorsman P 2010 γ-aminobutyric acid (GABA) Is an autocrine Excitatory Transmitter in Human Pancreatic β-Cells. Diabetes 59 1694–1701. (10.2337/db09-0797) 20413510 PMC2889769

[bib16] Braun M Wendt A Birnir B Broman J Eliasson L Galvanovskis J Gromada J Mulder H & Rorsman P 2004 Regulated exocytosis of GABA-containing synaptic-like microvesicles in pancreatic beta-cells. Journal of General Physiology 123 191–204. (10.1085/jgp.200308966) 14769845 PMC2217446

[bib17] Braun M Wendt A Karanauskaite J Galvanovskis J Clark A MacDonald PE & Rorsman P 2007 Corelease and differential exit via the fusion pore of GABA, serotonin, and ATP from LDCV in rat pancreatic beta cells. Journal of General Physiology 129 221–231. (10.1085/jgp.200609658) 17296927 PMC2151613

[bib18] Brereton MF Vergari E Zhang Q & Clark A 2015 Alpha-, delta- and PP-cells: are they the architectural cornerstones of islet structure and co-ordination? Journal of Histochemistry and Cytochemistry 63 575–591. (10.1369/0022155415583535) 26216135 PMC4530398

[bib19] Briant LJB Dodd MS Chibalina MV Rorsman NJG Johnson PRV Carmeliet P Rorsman P & Knudsen JG 2018 CPT1a-dependent long-chain fatty acid oxidation contributes to maintaining glucagon secretion from pancreatic islets. Cell Reports 23 3300–3311. (10.1016/j.celrep.2018.05.035) 29898400 PMC6581793

[bib20] Bru-Tari E Cobo-Vuilleumier N Alonso-Magdalena P Dos Santos RS Marroqui L Nadal A Gauthier BR & Quesada I 2019 Pancreatic alpha-cell mass in the early-onset and advanced stage of a mouse model of experimental autoimmune diabetes. Scientific Reports 9 9515. (10.1038/s41598-019-45853-1) PMC6606577 31266981

[bib21] Cabrera O Jacques-Silva MC Speier S Yang SN Köhler M Fachado A Vieira E Zierath JR Kibbey R Berman DM, 2008 Glutamate is a positive autocrine signal for glucagon release. Cell Metabolism 7 545–554. (10.1016/j.cmet.2008.03.004) 18522835 PMC4396785

[bib22] Campbell-Thompson M Butterworth EA Boatwright JL Nair MA Nasif LH Nasif K Revell AY Riva A Mathews CE Gerling IC, 2021 Islet sympathetic innervation and islet neuropathology in patients with type 1 diabetes. Scientific Reports 11 6562. (10.1038/s41598-021-85659-8) PMC7985489 33753784

[bib23] Chan O Cheng H Herzog R Czyzyk D Zhu W Wang A McCrimmon RJ Seashore MR & Sherwin RS 2008 Increased GABAergic tone in the ventromedial hypothalamus contributes to suppression of counterregulatory responses after antecedent hypoglycemia. Diabetes 57 1363–1370. (10.2337/db07-1559) 18375441 PMC5518793

[bib24] Cheng-Xue R Gomez-Ruiz A Antoine N Noel LA Chae HY Ravier MA Chimienti F Schuit FC & Gilon P 2013 Tolbutamide controls glucagon release from mouse islets differently than glucose: involvement of K(ATP) channels from both alpha-cells and delta-cells. Diabetes 62 1612–1622. (10.2337/db12-0347) 23382449 PMC3636641

[bib25] Collins SC Salehi A Eliasson L Olofsson CS & Rorsman P 2008 Long-term exposure of mouse pancreatic islets to oleate or palmitate results in reduced glucose-induced somatostatin and oversecretion of glucagon. Diabetologia 51 1689–1693. (10.1007/s00125-008-1082-0) 18622593 PMC2516194

[bib26] Coons AH Leduc EH & Connolly JM 1955 Studies on antibody production. I. A method for the histochemical demonstration of specific antibody and its application to a study of the hyperimmune rabbit. Journal of Experimental Medicine 102 49–60. (10.1084/jem.102.1.49) 14392240 PMC2136491

[bib27] Cryer PE 2006 Mechanisms of sympathoadrenal failure and hypoglycemia in diabetes. Journal of Clinical Investigation 116 1470–1473. (10.1172/JCI28735) 16741570 PMC1464914

[bib28] Cryer PE 2012 Minireview: glucagon in the pathogenesis of hypoglycemia and hyperglycemia in diabetes. Endocrinology 153 1039–1048. (10.1210/en.2011-1499) 22166985 PMC3281526

[bib29] Cryer PE & Gerich JE 1985 Glucose counterregulation, hypoglycemia, and intensive insulin therapy in diabetes mellitus. New England Journal of Medicine 313 232–241. (10.1056/NEJM198507253130405) 2861565

[bib30] Dadi PK Luo B Vierra NC & Jacobson DA 2015 TASK-1 potassium channels limit pancreatic alpha-cell calcium influx and glucagon secretion. Molecular Endocrinology 29 777–787. (10.1210/me.2014-1321) 25849724 PMC4415209

[bib31] Dai XQ Camunas-Soler J Briant LJB Dos Santos T Spigelman AF Walker EM Arrojo E Drigo R Bautista A Jones RC Avrahami D, 2022 Heterogenous impairment of alpha cell function in type 2 diabetes is linked to cell maturation state. Cell Metabolism 34 256–268.e5. (10.1016/j.cmet.2021.12.021) PMC8852281 35108513

[bib32] Davies SL Brown PD & Best L 2007 Glucose-induced swelling in rat pancreatic alpha-cells. Molecular and Cellular Endocrinology 264 61–67. (10.1016/j.mce.2006.10.005) 17112656

[bib33] De Marinis YZ Salehi A Ward CE Zhang Q Abdulkader F Bengtsson M Braha O Braun M Ramracheya R & Amisten S 2010 GLP-1 inhibits and adrenaline stimulates glucagon release by differential modulation of N-and L-type Ca^2+^ channel-dependent exocytosis. Cell Metabolism 11 543–553. (10.1016/j.cmet.2010.04.007) 20519125 PMC4310935

[bib34] DiGruccio MR Mawla AM Donaldson CJ Noguchi GM Vaughan J Cowing-Zitron C van der Meulen T & Huising MO 2016 Comprehensive alpha, beta and delta cell transcriptomes reveal that ghrelin selectively activates delta cells and promotes somatostatin release from pancreatic islets. Molecular Metabolism 5 449–458. (10.1016/j.molmet.2016.04.007) 27408771 PMC4921781

[bib35] Dolenšek J Rupnik MS & Stožer A 2015 Structural similarities and differences between the human and the mouse pancreas. Islets 7 e1024405. (10.1080/19382014.2015.1024405) PMC4589993 26030186

[bib36] Dunning BE & Gerich JE 2007 The role of alpha-cell dysregulation in fasting and postprandial hyperglycemia in type 2 diabetes and therapeutic implications. Endocrine Reviews 28 253–283. (10.1210/er.2006-0026) 17409288

[bib37] Edlund A Pedersen MG Lindqvist A Wierup N Flodström-Tullberg M & Eliasson L 2017 CFTR is involved in the regulation of glucagon secretion in human and rodent alpha cells. Scientific Reports 7 90. (10.1038/s41598-017-00098-8) PMC5428348 28273890

[bib38] Elliott AD Ustione A & Piston DW 2015 Somatostatin and insulin mediate glucose-inhibited glucagon secretion in the pancreatic alpha-cell by lowering cAMP. American Journal of Physiology. Endocrinology and Metabolism 308 E130–E143. (10.1152/ajpendo.00344.2014) 25406263 PMC4297778

[bib39] Elrick H Witten TA & Arai Y 1958 Glucagon treatment of insulin reactions. New England Journal of Medicine 258 476–480. (10.1056/NEJM195803062581005) 13517504

[bib40] Elrick MM Samson WK Corbett JA Salvatori AS Stein LM Kolar GR Naatz A & Yosten GL 2016 Neuronostatin acts via GPR107 to increase cAMP-independent PKA phosphorylation and proglucagon mRNA accumulation in pancreatic alpha-cells. American Journal of Physiology. Regulatory, Integrative and Comparative Physiology 310 R143–R155. (10.1152/ajpregu.00369.2014) 26561648 PMC4796643

[bib41] Faber CL Deem JD Campos CA Taborsky GJ & Morton GJ 2020 CNS control of the endocrine pancreas. Diabetologia 63 2086–2094. (10.1007/s00125-020-05204-6) 32894319 PMC7983553

[bib42] Flanagan DE Keshavarz T Evans ML Flanagan S Fan X Jacob RJ & Sherwin RS 2003 Role of corticotrophin-releasing hormone in the impairment of counterregulatory responses to hypoglycemia. Diabetes 52 605–613. (10.2337/diabetes.52.3.605) 12606499

[bib43] Franklin I Gromada J Gjinovci A Theander S & Wollheim CB 2005 Beta-cell secretory products activate alpha-cell ATP-dependent potassium channels to inhibit glucagon release. Diabetes 54 1808–1815. (10.2337/diabetes.54.6.1808) 15919803

[bib44] Furuyama K Chera S Van Gurp L Oropeza D Ghila L Damond N Vethe H Paulo JA Joosten AM Berney T, 2019 Diabetes relief in mice by glucose-sensing insulin-secreting human alpha-cells. Nature 567 43–48. (10.1038/s41586-019-0942-8) 30760930 PMC6624841

[bib45] Galvin SG Kay RG Foreman R Larraufie P Meek CL Biggs E Ravn P Jermutus L Reimann F & Gribble FM 2021 The human and mouse islet peptidome: effects of obesity and Type 2 diabetes, and assessment of intraislet production of glucagon-like Peptide-1. Journal of Proteome Research 20 4507–4517. (10.1021/acs.jproteome.1c00463) 34423991 PMC8419866

[bib46] Gerich JE Charles MA & Grodsky GM 1974 Characterization of the effects of arginine and glucose on glucagon and insulin release from the perfused rat pancreas. Journal of Clinical Investigation 54 833–841. (10.1172/JCI107823) 4430717 PMC301623

[bib47] Gloyn AL Pearson ER Antcliff JF Proks P Bruining GJ Slingerland AS Howard N Srinivasan S Silva JM Molnes J, 2004 Activating mutations in the gene encoding the ATP-sensitive potassium-channel subunit Kir6.2 and permanent neonatal diabetes. New England Journal of Medicine 350 1838–1849. (10.1056/NEJMoa032922) 15115830

[bib48] Gloyn AL Weedon MN Owen KR Turner MJ Knight BA Hitman G Walker M Levy JC Sampson M Halford S, 2003 Large-scale association studies of variants in genes encoding the pancreatic beta-cell KATP channel subunits Kir6.2 (KCNJ11) and SUR1 (ABCC8) confirm that the KCNJ11 E23K variant is associated with type 2 diabetes. Diabetes 52 568–572. (10.2337/diabetes.52.2.568) 12540637

[bib49] Göke B 2008 Islet cell function: alpha and beta cells--partners towards normoglycaemia. International Journal of Clinical Practice. Supplement (159) 2–7. (10.1111/j.1742-1241.2007.01686.x) 18269435

[bib50] Göpel S Kanno T Barg S Galvanovskis J & Rorsman P 1999 Voltage-gated and resting membrane currents recorded from B-cells in intact mouse pancreatic islets. Journal of Physiology 521 717–728. (10.1111/j.1469-7793.1999.00717.x) 10601501 PMC2269694

[bib51] Göpel S Zhang Q Eliasson L Ma XS Galvanovskis J Kanno T Salehi A & Rorsman P 2004 Capacitance measurements of exocytosis in mouse pancreatic alpha-, beta- and delta-cells within intact islets of Langerhans. Journal of Physiology 556 711–726. (10.1113/jphysiol.2003.059675) 14966302 PMC1664984

[bib52] Göpel SO Kanno T Barg S Weng XG Gromada J & Rorsman P 2000 Regulation of glucagon release in mouse -cells by KATP channels and inactivation of TTX-sensitive Na^+^ channels. Journal of Physiology 528 509–520. (10.1111/j.1469-7793.2000.00509.x) 11060128 PMC2270147

[bib53] Gromada J Bokvist K Ding WG Barg S Buschard K Renström E & Rorsman P 1997 Adrenaline stimulates glucagon secretion in pancreatic A-cells by increasing the Ca^2+^ current and the number of granules close to the L-type Ca^2+^ channels. Journal of General Physiology 110 217–228. (10.1085/jgp.110.3.217) 9276750 PMC2229364

[bib54] Gromada J Hoy M Buschard K Salehi A & Rorsman P 2001 Somatostatin inhibits exocytosis in rat pancreatic alpha-cells by G(i2)-dependent activation of calcineurin and depriming of secretory granules. Journal of Physiology 535 519–532. (10.1111/j.1469-7793.2001.00519.x) 11533141 PMC2278803

[bib55] Gromada J Ma X Hoy M Bokvist K Salehi A Berggren PO & Rorsman P 2004 ATP-sensitive K^+^ channel-dependent regulation of glucagon release and electrical activity by glucose in wild-type and SUR1-/- mouse alpha-cells. Diabetes 53(Supplement 3) S181–S189. (10.2337/diabetes.53.suppl_3.s181) 15561909

[bib56] Guenat E Seematter G Philippe J Temler E Jequier E & Tappy L 2000 Counterregulatory responses to hypoglycemia in patients with glucokinase gene mutations. Diabetes and Metabolism 26 377–384. 11119017

[bib57] Gylfe E 2016 Glucose control of glucagon secretion-'There's a brand-new gimmick every year'. Upsala Journal of Medical Sciences 121 120–132. (10.3109/03009734.2016.1154905) 27044660 PMC4900067

[bib58] Hamill OP Marty A Neher E Sakmann B & Sigworth FJ 1981 Improved patch-clamp techniques for high-resolution current recording from cells and cell-free membrane patches. Pflugers Archiv 391 85–100. (10.1007/BF00656997) 6270629

[bib59] Hamilton A Zhang Q Salehi A Willems M Knudsen JG Ringgaard AK Chapman CE Gonzalez-Alvarez A Surdo NC Zaccolo M, 2018 Adrenaline stimulates glucagon secretion by Tpc2-dependent Ca^2+^ mobilization from acidic stores in pancreatic α-cells. Diabetes 67 1128–1139. (10.2337/db17-1102) 29563152 PMC6258900

[bib60] Hardy AB Serino AS Wijesekara N Chimienti F & Wheeler MB 2011 Regulation of glucagon secretion by zinc: lessons from the beta cell-specific Znt8 knockout mouse model. Diabetes, Obesity and Metabolism 13(Supplement 1) 112–117. (10.1111/j.1463-1326.2011.01451.x) 21824264

[bib61] Heimberg H De Vos A Pipeleers D Thorens B & Schuit F 1995 Differences in glucose transporter Gene Expression between Rat Pancreatic α- and β-Cells Are Correlated to Differences in glucose Transport but Not in glucose Utilization*. Journal of Biological Chemistry 270 8971–8975. (10.1074/jbc.270.15.8971) 7721807

[bib62] Heller SR & Cryer PE 1991 Reduced neuroendocrine and symptomatic responses to subsequent hypoglycemia after 1 episode of hypoglycemia in nondiabetic humans. Diabetes 40 223–226. (10.2337/diab.40.2.223) 1991573

[bib63] Huang WQ Guo JH Zhang XH Yu MK Chung YW Ruan YC & Chan HC 2017 Glucose-Sensitive CFTR Suppresses glucagon Secretion by Potentiating KATP Channels in Pancreatic Islet alpha Cells. Endocrinology 158 3188–3199. (10.1210/en.2017-00282) 28977595

[bib64] Huang YC Rupnik MS Karimian N Herrera PL Gilon P Feng ZP & Gaisano HY 2013 In situ electrophysiological examination of pancreatic alpha cells in the streptozotocin-induced diabetes model, revealing the cellular basis of glucagon hypersecretion. Diabetes 62 519–530. (10.2337/db11-0786) 23043159 PMC3554363

[bib65] Hughes JW Ustione A Lavagnino Z & Piston DW 2018 Regulation of islet glucagon secretion: beyond calcium. Diabetes, Obesity and Metabolism 20(Supplement 2) 127–136. (10.1111/dom.13381) PMC6148361 30230183

[bib66] Hutchens T & Piston DW 2015 EphA4 receptor forward signaling inhibits glucagon secretion from alpha-cells. Diabetes 64 3839–3851. (10.2337/db15-0488) 26251403 PMC4613968

[bib67] Ikeuchi M & Yagi K 1982 Pancreatic A cell generates action potential. Japanese Journal of Physiology 32 873–878. (10.2170/jjphysiol.32.873) 6759738

[bib68] Ishihara H Maechler P Gjinovci A Herrera PL & Wollheim CB 2003 Islet beta-cell secretion determines glucagon release from neighbouring alpha-cells. Nature Cell Biology 5 330–335. (10.1038/ncb951) 12640462

[bib69] Kailey B Van De Bunt M Cheley S Johnson PR MacDonald PE Gloyn AL Rorsman P & Braun M 2012 SSTR2 is the functionally dominant somatostatin receptor in human pancreatic beta- and alpha-cells. American Journal of Physiology. Endocrinology and Metabolism 303 E1107–E1116. (10.1152/ajpendo.00207.2012) 22932785 PMC3492856

[bib70] Kaneko K Shirotani T Araki E Matsumoto K Taguchi T Motoshima H Yoshizato K Kishikawa H & Shichiri M 1999 Insulin inhibits glucagon secretion by the activation of PI3-kinase in In-R1-G9 cells. Diabetes Research and Clinical Practice 44 83–92. (10.1016/s0168-8227(9900021-2) 10414926

[bib71] Karimian N Qin T Liang T Osundiji M Huang Y Teich T Riddell MC Cattral MS Coy DH Vranic M, 2013 Somatostatin receptor type 2 antagonism improves glucagon counterregulation in biobreeding diabetic rats. Diabetes 62 2968–2977. (10.2337/db13-0164) 23630299 PMC3717832

[bib72] Kawamori D Kurpad AJ Hu J Liew CW Shih JL Ford EL Herrera PL Polonsky KS Mcguinness OP & Kulkarni RN 2009 Insulin signaling in alpha cells modulates glucagon secretion in vivo. Cell Metabolism 9 350–361. (10.1016/j.cmet.2009.02.007) 19356716 PMC2694613

[bib73] Keighron JD Wigström J Kurczy ME Bergman J Wang Y & Cans AS 2015 Amperometric detection of single vesicle acetylcholine release events from an artificial cell. ACS Chemical Neuroscience 6 181–188. (10.1021/cn5002667) 25565357

[bib74] Kimball CP & Murlin JR 1923 Aqueous extracts of pancreas: III. Journal of Biological Chemistry 58 337–346. (10.1016/S0021-9258(1885474-6)

[bib75] Knudsen JG Hamilton A Ramracheya R Tarasov AI Brereton M Haythorne E Chibalina MV Spégel P Mulder H Zhang Q, 2019 Dysregulation of glucagon secretion by hyperglycemia-induced sodium-dependent reduction of ATP production. Cell Metabolism 29 430–442.e4. (10.1016/j.cmet.2018.10.003) 30415925 PMC6370947

[bib76] Krippeit-Drews P Backer M Dûfer M & Drews G 2003 Phosphocreatine as a determinant of K(ATP) channel activity in pancreatic beta-cells. Pflugers Archiv 445 556–562. (10.1007/s00424-002-0975-x) 12634926

[bib77] Lai BK Chae H Gomez-Ruiz A Cheng P Gallo P Antoine N Beauloye C Jonas JC Seghers V Seino S, 2018 Somatostatin Is Only Partly Required for the Glucagonostatic Effect of Glucose but Is Necessary for the Glucagonostatic Effect of KATP Channel Blockers. Diabetes 67 2239–2253. (10.2337/db17-0880) 30115649

[bib78] Lane MA 1907 The cytological characters of the areas of langerhans. American Journal of Anatomy 7 409–422. (10.1002/aja.1000070304)

[bib79] Le Marchand SJ & Piston DW 2012 Glucose decouples intracellular Ca2+ activity from glucagon secretion in mouse pancreatic islet alpha-cells. PLoS One 7 e47084. (10.1371/journal.pone.0047084) PMC3471958 23077547

[bib80] Leibiger B Moede T Muhandiramlage TP Kaiser D Vaca Sanchez P Leibiger IB & Berggren PO 2012 Glucagon regulates its own synthesis by autocrine signaling. Proceedings of the National Academy of Sciences 109 20925–20930. (10.1073/pnas.1212870110) PMC3529083 23213228

[bib81] Leung YM Ahmed I Sheu L Gao X Hara M Tsushima RG Diamant NE & Gaisano HY 2006 Insulin regulates islet alpha-cell function by reducing KATP channel sensitivity to adenosine 5'-triphosphate inhibition. Endocrinology 147 2155–2162. (10.1210/en.2005-1249) 16455778

[bib82] Li C Liu C Nissim I Chen J Chen P Doliba N Zhang T Nissim I Daikhin Y Stokes D, 2013 Regulation of glucagon secretion in normal and diabetic human islets by gamma-hydroxybutyrate and glycine. Journal of Biological Chemistry 288 3938–3951. (10.1074/jbc.M112.385682) 23266825 PMC3567647

[bib83] Liu YJ Vieira E & Gylfe E 2004 A store-operated mechanism determines the activity of the electrically excitable glucagon-secreting pancreatic alpha-cell. Cell Calcium 35 357–365. (10.1016/j.ceca.2003.10.002) 15036952

[bib84] Ma X Zhang Y Gromada J Sewing S Berggren PO Buschard K Salehi A Vikman J Rorsman P & Eliasson L 2005 Glucagon stimulates exocytosis in mouse and rat pancreatic alpha-cells by binding to glucagon receptors. Molecular Endocrinology 19 198–212. (10.1210/me.2004-0059) 15459251

[bib85] MacDonald PE De Marinis YZ Ramracheya R Salehi A Ma X Johnson PR Cox R Eliasson L & Rorsman P 2007 A K ATP channel-dependent pathway within alpha cells regulates glucagon release from both rodent and human islets of Langerhans. PLOS Biology 5 e143. (10.1371/journal.pbio.0050143) PMC1868042 17503968

[bib86] Marchetti P Lupi R Bugliani M Kirkpatrick CL Sebastiani G Grieco FA Del Guerra S D'aleo V Piro S Marselli L, 2012 A local glucagon-like peptide 1 (GLP-1) system in human pancreatic islets. Diabetologia 55 3262–3272. (10.1007/s00125-012-2716-9) 22965295

[bib87] McCrimmon RJ Evans ML Fan X Mcnay EC Chan O Ding Y Zhu W Gram DX & Sherwin RS 2005 Activation of ATP-sensitive K+ channels in the ventromedial hypothalamus amplifies counterregulatory hormone responses to hypoglycemia in normal and recurrently hypoglycemic rats. Diabetes 54 3169–3174. (10.2337/diabetes.54.11.3169) 16249441

[bib88] McKinnon CM Ravier MA & Rutter GA 2006 FoxO1 is required for the regulation of preproglucagon gene expression by insulin in pancreatic alphaTC1-9 cells. Journal of Biological Chemistry 281 39358–39369. (10.1074/jbc.M605022200) 17062568

[bib89] Menegaz D Hagan DW Almaca J Cianciaruso C Rodriguez-Diaz R Molina J Dolan RM Becker MW Schwalie PC Nano R, 2019 Mechanism and effects of pulsatile GABA secretion from cytosolic pools in the human beta cell. Nature Metabolism 1 1110–1126. (10.1038/s42255-019-0135-7) PMC7236889 32432213

[bib90] Menge BA Grûber L Jørgensen SM Deacon CF Schmidt WE Veldhuis JD Holst JJ & Meier JJ 2011 Loss of inverse relationship between pulsatile insulin and glucagon secretion in patients with type 2 diabetes. Diabetes 60 2160–2168. (10.2337/db11-0251) 21677283 PMC3142077

[bib91] Milescu LS Yamanishi T Ptak K Mogri MZ & Smith JC 2008 Real-time kinetic modeling of voltage-gated ion channels using dynamic clamp. Biophysical Journal 95 66–87. (10.1529/biophysj.107.118190) 18375511 PMC2426646

[bib92] Molina J Rodriguez-Diaz R Fachado A Jacques-Silva MC Berggren PO & Caicedo A 2014 Control of insulin secretion by cholinergic signaling in the human pancreatic islet. Diabetes 63 2714–2726. (10.2337/db13-1371) 24658304 PMC4113066

[bib93] Mundinger TO & Taborsky GJ 2016 Early sympathetic islet neuropathy in autoimmune diabetes: lessons learned and opportunities for investigation. Diabetologia 59 2058–2067. (10.1007/s00125-016-4026-0) 27342407 PMC6214182

[bib94] Murlin JR Clough HD Gibbs CBF & Stokes AM 1923 Aqueous extracts of pancreas: I. Journal of Biological Chemistry 56 253–296. (10.1016/S0021-9258(1885619-8)

[bib95] Nakamura A & Terauchi Y 2015 Present status of clinical deployment of glucokinase activators. Journal of Diabetes Investigation 6 124–132. (10.1111/jdi.12294) 25802718 PMC4364845

[bib96] Ng XW Chung YH Asadi F Kong C Ustione A & Piston DW 2022 RhoA as a signaling hub controlling glucagon secretion from pancreatic alpha-cells. Diabetes 71 2384–2394. (10.2337/db21-1010) 35904939 PMC9630081

[bib97] Nicolson TJ Bellomo EA Wijesekara N Loder MK Baldwin JM Gyulkhandanyan AV Koshkin V Tarasov AI Carzaniga R Kronenberger K, 2009 Insulin storage and glucose homeostasis in mice null for the granule zinc transporter ZnT8 and studies of the type 2 diabetes-associated variants. Diabetes 58 2070–2083. (10.2337/db09-0551) 19542200 PMC2731533

[bib98] Nordenskjöld F, Andersson B & Islam MS 2020 Expression of the inositol 1,4,5-trisphosphate receptor and the ryanodine receptor Ca2+-release channels in the beta-cells and alpha-cells of the human islets of Langerhans. In Islam MS, Ed. Calcium Signaling. Cham: Springer International Publishing 271–279. (10.1007/978-3-030-12457-1_11) 31646514

[bib99] Okamoto H Cavino K Na E Krumm E Kim SY Cheng X Murphy AJ Yancopoulos GD & Gromada J 2017 Glucagon receptor inhibition normalizes blood glucose in severe insulin-resistant mice. Proceedings of the National Academy of Sciences of the United States of America 114 2753–2758. (10.1073/pnas.1621069114) 28115707 PMC5347550

[bib100] Omar-Hmeadi M Lund PE Gandasi NR Tengholm A & Barg S 2020 Paracrine control of α-cell glucagon exocytosis is compromised in human type-2 diabetes. Nature Communications 11 1896. (10.1038/s41467-020-15717-8) PMC7171169 32312960

[bib101] Pettus J Reeds D Cavaiola TS Boeder S Levin M Tobin G Cava E Thai D Shi J Yan H, 2018 Effect of a glucagon receptor antibody (REMD-477) in type 1 diabetes: a randomized controlled trial. Diabetes, Obesity and Metabolism 20 1302–1305. (10.1111/dom.13202) PMC6181222 29283470

[bib102] Pfeifer CR Shomorony A Aronova MA Zhang G Cai T Xu H Notkins AL & Leapman RD 2015 Quantitative analysis of mouse pancreatic islet architecture by serial block-face SEM. Journal of Structural Biology 189 44–52. (10.1016/j.jsb.2014.10.013) 25448885 PMC4305430

[bib103] Pizarro-Delgado J Braun M Hernandez-Fisac I Martin-Del-Rio R & Tamarit-Rodriguez J 2010 Glucose promotion of GABA metabolism contributes to the stimulation of insulin secretion in beta-cells. Biochemical Journal 431 381–389. (10.1042/BJ20100714) 20695849

[bib104] Quoix N Cheng-Xue R Mattart L Zeinoun Z Guiot Y Beauvois MC Henquin JC & Gilon P 2009 Glucose and pharmacological modulators of ATP-sensitive K+ channels control [Ca2+]c by different mechanisms in isolated mouse alpha-cells. Diabetes 58 412–421. (10.2337/db07-1298) 19008345 PMC2628615

[bib105] Raju B & Cryer PE 2005 Loss of the decrement in intraislet insulin plausibly explains loss of the glucagon response to hypoglycemia in insulin-deficient diabetes: documentation of the intraislet insulin hypothesis in humans. Diabetes 54 757–764. (10.2337/diabetes.54.3.757) 15734853

[bib106] Ramracheya R Ward C Shigeto M Walker JN Amisten S Zhang Q Johnson PR Rorsman P & Braun M 2010 Membrane potential-dependent inactivation of voltage-gated ion channels in α-cells inhibits glucagon secretion from human islets. Diabetes 59 2198–2208. (10.2337/db09-1505) 20547976 PMC2927942

[bib107] Ravier MA & Rutter GA 2005 Glucose or insulin, but not zinc ions, inhibit glucagon secretion from mouse pancreatic alpha-cells. Diabetes 54 1789–1797. (10.2337/diabetes.54.6.1789) 15919801

[bib108] Rodriguez-Diaz R Dando R Jacques-Silva MC Fachado A Molina J Abdulreda MH Ricordi C Roper SD Berggren PO & Caicedo A 2011b Alpha cells secrete acetylcholine as a non-neuronal paracrine signal priming beta cell function in humans. Nature Medicine 17 888–892. (10.1038/nm.2371) PMC3132226 21685896

[bib109] Rodriguez-Diaz R Abdulreda MH Formoso AL Gans I Ricordi C Berggren PO & Caicedo A 2011a Innervation patterns of autonomic axons in the human endocrine pancreas. Cell Metabolism 14 45–54. (10.1016/j.cmet.2011.05.008) 21723503 PMC3135265

[bib110] Rorsman P 1988 Two types of Ca^2+^ currents with different sensitivities to organic Ca^2+^ channel antagonists in guinea pig pancreatic alpha 2 cells. Journal of General Physiology 91 243–254. (10.1085/jgp.91.2.243) 2453604 PMC2216126

[bib111] Rorsman P Berggren PO Bokvist K Ericson H Möhler H Östenson CG & Smith PA 1989 Glucose-inhibition of glucagon secretion involves activation of GABA_A_-receptor chloride channels. Nature 341 233–236. (10.1038/341233a0) 2550826

[bib112] Rorsman P & Hellman B 1988 Voltage-activated currents in guinea pig pancreatic alpha 2 cells. Evidence for Ca^2+^-dependent action potentials. Journal of General Physiology 91 223–242. (10.1085/jgp.91.2.223) 2453603 PMC2216127

[bib113] Samson WK Zhang JV Avsian-Kretchmer O Cui K Yosten GL Klein C Lyu RM Wang YX Chen XQ Yang J, 2008 Neuronostatin encoded by the somatostatin gene regulates neuronal, cardiovascular, and metabolic functions. Journal of Biological Chemistry 283 31949–31959. (10.1074/jbc.M804784200) 18753129 PMC2581552

[bib114] Saponaro C Spiliotis I Acosta-Montalvo A Anguelova L Thevenet J Chiral M Gmyr V Kerr-Conte J Pattou F Pontoglio M, 2022 Low-dose treatment with sulfonylureas improves hyperglucagonemia after a glucose challenge in HNF1A-MODY individuals and Hnf1a-/- mice. Diabetologia 65 S162.

[bib115] Sato Y Rahman MM Haneda M Tsuyama T Mizumoto T Yoshizawa T Kitamura T Gonzalez FJ Yamamura KI & Yamagata K 2020 HNF1alpha controls glucagon secretion in pancreatic alpha-cells through modulation of SGLT1. Biochimica et Biophysica Acta. Molecular Basis of Disease 1866 165898. (10.1016/j.bbadis.2020.165898) PMC8865093 32711050

[bib116] Schwanstecher C Meyer U & Schwanstecher M 2002 K(IR)6.2 polymorphism predisposes to type 2 diabetes by inducing overactivity of pancreatic beta-cell ATP-sensitive K(+) channels. Diabetes 51 875–879. (10.2337/diabetes.51.3.875) 11872696

[bib117] Schwartz NS Clutter WE Shah SD & Cryer PE 1987 Glycemic thresholds for activation of glucose counterregulatory systems are higher than the threshold for symptoms. Journal of Clinical Investigation 79 777–781. (10.1172/JCI112884) 3546378 PMC424197

[bib118] Shah P Vella A Basu A Basu R Schwenk WF & Rizza RA 2000 Lack of suppression of glucagon contributes to postprandial hyperglycemia in subjects with type 2 diabetes mellitus. Journal of Clinical Endocrinology and Metabolism 85 4053–4059. (10.1210/jcem.85.11.6993) 11095432

[bib119] Sherwin RS 2008 Bringing light to the dark side of insulin: a journey across the blood-brain barrier. Diabetes 57 2259–2268. (10.2337/db08-9023) 18753671 PMC2518475

[bib120] Singh B Khattab F Chae H Desmet L Herrera PL & Gilon P 2021 K(ATP) channel blockers control glucagon secretion by distinct mechanisms: a direct stimulation of alpha-cells involving a [Ca(2+)](c) rise and an indirect inhibition mediated by somatostatin. Molecular Metabolism 53 101268. (10.1016/j.molmet.2021.101268) PMC8274344 34118477

[bib121] Slucca M Harmon JS Oseid EA Bryan J & Robertson RP 2010 ATP-sensitive K+ channel mediates the zinc switch-off signal for glucagon response during glucose deprivation. Diabetes 59 128–134. (10.2337/db09-1098) 19808893 PMC2797913

[bib122] Spigelman AF Dai X & MacDonald PE 2010 Voltage-dependent K^+^ channels are positive regulators of alpha cell action potential generation and glucagon secretion in mice and humans. Diabetologia 53 1917–1926. (10.1007/s00125-010-1759-z) 20446079

[bib123] Spiliotis II Chalk R Gough S. & Rorsman P 2022 Reducing hyperglucagonaemia in type 2 diabetes using low-dose glibenclamide: results of the Legend-A pilot study. Diabetes, Obesity and Metabolism 24 1671–1675. (10.1111/dom.14740) PMC9543075 35491519

[bib124] Staub A Sinn L & Behrens OK 1953 Purification and crystallization of hyperglycemic glycogenolytic factor (HGF). Science 117 628–629. (10.1126/science.117.3049.628) 13056638

[bib125] Steenberg VR Jensen SM Pedersen J Madsen AN Windeløv JA Holst B Quistorff B Poulsen SS & Holst JJ 2016 Acute disruption of glucagon secretion or action does not improve glucose tolerance in an insulin-deficient mouse model of diabetes. Diabetologia 59 363–370. (10.1007/s00125-015-3794-2) 26537124

[bib126] Suga T Kikuchi O Kobayashi M Matsui S Yokota-Hashimoto H Wada E Kohno D Sasaki T Takeuchi K Kakizaki S, 2019 SGLT1 in pancreatic alpha cells regulates glucagon secretion in mice, possibly explaining the distinct effects of SGLT2 inhibitors on plasma glucagon levels. Molecular Metabolism 19 1–12. (10.1016/j.molmet.2018.10.009) 30416006 PMC6323192

[bib127] Sutherland EW & De Duve C 1948 Origin and distribution of the hyperglycemic-glycogenolytic factor of the pancreas. Journal of Biological Chemistry 175 663–674. (10.1016/S0021-9258(1857183-0) 18880761

[bib128] Taborsky GJ Mundinger TO 2012 Minireview: the role of the autonomic nervous system in mediating the glucagon response to hypoglycemia. Endocrinology 153 1055–1062. (10.1210/en.2011-2040) 22315452 PMC3384078

[bib129] Taleb N & Rabasa-Lhoret R 2016 Can somatostatin antagonism prevent hypoglycaemia during exercise in type 1 diabetes? Diabetologia 59 1632–1635. (10.1007/s00125-016-3978-4) 27153841

[bib130] Taneera J Jin Z Jin Y Muhammed SJ Zhang E Lang S Salehi A Korsgren O Renström E Groop L, 2012 Gamma-aminobutyric acid (GABA) signalling in human pancreatic islets is altered in type 2 diabetes. Diabetologia 55 1985–1994. (10.1007/s00125-012-2548-7) 22538358 PMC3369140

[bib131] Tang SC Baeyens L Shen CN Peng SJ Chien HJ Scheel DW Chamberlain CE & German MS 2018 Human pancreatic neuro-insular network in health and fatty infiltration. Diabetologia 61 168–181. (10.1007/s00125-017-4409-x) 28852792

[bib132] Thomas-Reetz A Hell JW During MJ Walch-Solimena C Jahn R & De Camilli P 1993 A gamma-aminobutyric acid transporter driven by a proton pump is present in synaptic-like microvesicles of pancreatic beta cells. Proceedings of the National Academy of Sciences of the United States of America 90 5317–5321. (10.1073/pnas.90.11.5317) 8506380 PMC46707

[bib133] Tschritter O Stumvoll M Machicao F Holzwarth M Weisser M Maerker E Teigeler A Haring H & Fritsche A 2002 The prevalent Glu23Lys polymorphism in the potassium inward rectifier 6.2 (KIR6.2) gene is associated with impaired glucagon suppression in response to hyperglycemia. Diabetes 51 2854–2860. (10.2337/diabetes.51.9.2854) 12196481

[bib134] Tsuchiyama N Takamura T Ando H Sakurai M Shimizu A Kato K Kurita S & Kaneko S 2007 Possible role of alpha-cell insulin resistance in exaggerated glucagon responses to arginine in type 2 diabetes. Diabetes Care 30 2583–2587. (10.2337/dc07-0066) 17644622

[bib135] Unger RH & Cherrington AD 2012 Glucagonocentric restructuring of diabetes: a pathophysiologic and therapeutic makeover. Journal of Clinical Investigation 122 4–12. (10.1172/JCI60016) 22214853 PMC3248306

[bib136] Unger RH Eisentraut AM Mccall MS Keller S Lanz HC & Madison LL 1959 Glucagon antibodies and their use for immunoassay for glucagon. Proceedings of the Society for Experimental Biology and Medicine. Society for Experimental Biology and Medicine 102 621–623. (10.3181/00379727-102-25338) 13840405

[bib137] van der Meulen T Donaldson CJ Cáceres E Hunter AE Cowing-Zitron C Pound LD Adams MW Zembrzycki A Grove KL & Huising MO 2015 Urocortin3 mediates somatostatin-dependent negative feedback control of insulin secretion. Nature Medicine 21 769–776. (10.1038/nm.3872) PMC4496282 26076035

[bib138] Veprik A Denwood G Liu D Bany Bakar R Morfin V Mchugh K Tebeka NN Vetterli L Yonova-Doing E Gribble F, 2022 Acetyl-CoA-carboxylase 1 (ACC1) plays a critical role in glucagon secretion. Communications Biology 5 238. (10.1038/s42003-022-03170-w) PMC8933412 35304577

[bib139] Vieira E Liu YJ & Gylfe E 2004 Involvement of α1 and β-adrenoceptors in adrenaline stimulation of the glucagon-secreting mouse α-cell. Naunyn-Schmiedeberg's Archives of Pharmacology 369 179–183. (10.1007/s00210-003-0858-5) 14727006

[bib140] Vieira E Salehi A & Gylfe E 2007 Glucose inhibits glucagon secretion by a direct effect on mouse pancreatic alpha cells. Diabetologia 50 370–379. (10.1007/s00125-006-0511-1) 17136393

[bib141] Wang C Kerckhofs K Van De Casteele M Smolders I Pipeleers D & Ling Z 2006 Glucose inhibits GABA release by pancreatic beta-cells through an increase in GABA shunt activity. American Journal of Physiology. Endocrinology and Metabolism 290 E494–E499. (10.1152/ajpendo.00304.2005) 16249254

[bib142] Wendt A Birnir B Buschard K Gromada J Salehi A Sewing S Rorsman P & Braun M 2004 Glucose inhibition of glucagon secretion from rat alpha-cells is mediated by GABA released from neighboring beta-cells. Diabetes 53 1038–1045. (10.2337/diabetes.53.4.1038) 15047619

[bib143] Wesslen N Pipeleers D Van De Winkel M Rorsman P & Hellman B 1987 Glucose stimulates the entry of Ca2+ into the insulin-producing beta cells but not into the glucagon-producing alpha 2 cells. Acta Physiologica Scandinavica 131 230–234. (10.1111/j.1748-1716.1987.tb08231.x) 3314351

[bib144] Xia F Leung YM Gaisano G Gao X Chen Y Fox JE Bhattacharjee A Wheeler MB Gaisano HY & Tsushima RG 2007 Targeting of voltage-gated K+ and Ca2+ channels and soluble N-ethylmaleimide-sensitive factor attachment protein receptor proteins to cholesterol-rich lipid rafts in pancreatic alpha-cells: effects on glucagon stimulus-secretion coupling. Endocrinology 148 2157–2167. (10.1210/en.2006-1296) 17303668

[bib145] Xin Y Kim J Okamoto H Ni M Wei Y Adler C Murphy AJ Yancopoulos GD Lin C & Gromada J 2016 RNA sequencing of single human islet cells reveals Type 2 diabetes genes. Cell Metabolism 24 608–615. (10.1016/j.cmet.2016.08.018) 27667665

[bib146] Xu E Kumar M Zhang Y Ju W Obata T Zhang N Liu S Wendt A Deng S Ebina Y, 2006 Intra-islet insulin suppresses glucagon release via GABA-GABAA receptor system. Cell Metabolism 3 47–58. (10.1016/j.cmet.2005.11.015) 16399504

[bib147] Xu SFS Andersen DB Izarzugaza JMG Kuhre RE & Holst JJ 2020 In the rat pancreas, somatostatin tonically inhibits glucagon secretion and is required for glucose-induced inhibition of glucagon secretion. Acta Physiologica (Oxford, England) 229 e13464. (10.1111/apha.13464) 32145704

[bib148] Yan-Do R Duong E Manning Fox JE Dai X Suzuki K Khan S Bautista A Ferdaoussi M Lyon J Wu X, 2016 A glycine-insulin autocrine feedback loop enhances insulin secretion from human beta-cells and is impaired in Type 2 diabetes. Diabetes 65 2311–2321. (10.2337/db15-1272) 27207556

[bib149] Yu Q Shuai H Ahooghalandari P Gylfe E & Tengholm A 2019 Glucose controls glucagon secretion by directly modulating cAMP in alpha cells. Diabetologia 62 1212–1224. (10.1007/s00125-019-4857-6) 30953108 PMC6560012

[bib150] Yue JTY Riddell MC Burdett E Coy DH Efendic S & Vranic M 2013 Amelioration of hypoglycemia via somatostatin receptor Type 2 antagonism in recurrently hypoglycemic diabetic rats. Diabetes 62 2215–2222. (10.2337/db12-1523) PMC3712070 23434929

[bib151] Zaborska KE Dadi PK Dickerson MT Nakhe AY Thorson AS Schaub CM Graff SM Stanley JE Kondapavuluru RS Denton JS, 2020 Lactate activation of alpha-cell KATP channels inhibits glucagon secretion by hyperpolarizing the membrane potential and reducing Ca(2+) entry. Molecular Metabolism 42 101056. (10.1016/j.molmet.2020.101056) PMC7479281 32736089

[bib152] Zhang Q Chibalina MV Bengtsson M Groschner LN Ramracheya R Rorsman NJ Leiss V Nassar MA Welling A Gribble FM, 2014 Na^+^ current properties in islet α‐and β‐cells reflect cell‐specific Scn3a and Scn9a expression. Journal of Physiology 592 4677–4696. (10.1113/jphysiol.2014.274209) 25172946 PMC4253470

[bib153] Zhang Q Ramracheya R Lahmann C Tarasov A Bengtsson M Braha O Braun M Brereton M Collins S Galvanovskis J, 2013 Role of KATP channels in glucose-regulated glucagon secretion and impaired counterregulation in type 2 diabetes. Cell Metabolism 18 871–882. (10.1016/j.cmet.2013.10.014) 24315372 PMC3851686

[bib154] Zhou H Zhang T Harmon JS Bryan J & Robertson RP 2007 Zinc, not insulin, regulates the rat alpha-cell response to hypoglycemia in vivo. Diabetes 56 1107–1112. (10.2337/db06-1454) 17317764

